# SRAF-ID: A Sensor-Reliability-Aware Framework for Robust Traffic Speed Forecasting Under Missing and Faulty Sensor Observations

**DOI:** 10.3390/s26165263

**Published:** 2026-08-19

**Authors:** Peng Lu, Daming Wu, Shaofei Lan, Beinan Guo, Zixiao Li

**Affiliations:** 1 School of Emergency Management Science and Engineering, University of Chinese Academy of Sciences, Beijing 100049, China; lupeng24@mails.ucas.ac.cn; 2 National Institute of Natural Hazards, Ministry of Emergency Management of the People’s Republic of China, Beijing 100085, China; lanshaofei24@mails.ucas.ac.cn (S.L.); 202131260004@mail.bnu.edu.cn (B.G.); zixiaoli@ninhm.ac.cn (Z.L.); 3 Key Laboratory of Compound and Chained Natural Hazards Dynamics, Ministry of Emergency Management of China, Beijing 100085, China

**Keywords:** traffic speed forecasting, traffic sensor faults, sensor data repair, sensor data reliability, missing data, spatiotemporal forecasting

## Abstract

Reliable traffic speed forecasting depends on trustworthy historical road-sensor observations, yet deployed sensors may exhibit missing values, outages, noise, calibration drift, and stuck readings. Existing forecasting models are commonly evaluated on cleaned inputs, whereas conventional imputation optimizes historical reconstruction rather than downstream prediction. This study presents the Sensor-Reliability-Aware Framework with Identity-Preserved Design (SRAF-ID), a prediction-oriented speed-channel repair front-end trained end to end using only future forecasting loss. The final model requires no controlled fault-location labels during training or inference. SRAF-ID constructs same-sensor temporal and mask-aware graph-neighborhood candidates, combines them through learned two-way softmax fusion, and preserves node-identity and temporal-context features. On raw-time-disjoint 70%/10%/20% splits of the Metropolitan Los Angeles (METR-LA) and California Performance Measurement System Bay Area (PEMS-BAY) datasets, ten-seed matched stress tests cover six window-level controlled perturbations. SRAF-ID reduces faulty-average mean absolute error from 5.12 to 4.82 on METR-LA and from 1.99 to 1.94 on PEMS-BAY, corresponding to relative reductions of 5.7% and 2.4%, respectively. It achieves a lower mean MAE in all 12 dataset-fault comparisons and a lower faulty-average MAE in all ten seeds on both datasets; the clean-input MAE also decreases. Checkpoint-only tests retain positive all-sensor and affected-sensor mean gains in all eight localized dataset-condition pairs, whereas unseen 0.75-standard-deviation global drift produces small adverse means with paired intervals crossing zero. The evidence therefore supports fault-label-free robustness under the defined stress protocols while leaving field-recorded event continuity and fault frequency for external validation.

## 1. Introduction

Traffic speed forecasting relies on continuous and trustworthy historical observations from road sensors. In deployed systems, however, measurements can be degraded by packet loss, power or communication outages, electrical interference, calibration drift, installation defects, and stuck electronics. Operational evidence indicates that these problems can reach consequential scales: Caltrans District 3 reported that approximately 20% of detectors were out of service on 1 April 2025, whereas an actively maintained Chicago network cited by the Federal Highway Administration kept the share of inoperative loop detectors below approximately 5% at any given time [1,2]. These figures are system- and period-specific rather than universal failure rates, but they demonstrate that detector availability and health cannot be assumed. Such degradation changes the historical-input distribution received by forecasting models and can destabilize future speed prediction. Short-term traffic forecasting is therefore important not only for intelligent transportation system operations, traffic control, and route guidance [3,4] but also for maintaining dependable decision support when sensing quality deteriorates.

A rich family of traffic forecasting methods has been developed. Early statistical and conventional machine-learning models provide interpretable short-term forecasting baselines but often struggle with nonlinear traffic dynamics. Deep learning models enhance temporal and spatial dependency learning through recurrent networks, temporal convolutions, graph neural networks, and attention mechanisms. DCRNN [5] combines diffusion convolution with recurrent modeling to capture diffusion processes on road networks; STGCN [6] jointly models spatio-temporal dependencies using graph and temporal convolutions; Graph WaveNet [7] introduces adaptive adjacency learning and dilated temporal convolution; GMAN [8] uses graph multi-attention to model spatial and temporal dependencies; AGCRN [9] improves sensor-specific modeling through node-adaptive parameters; and PDFormer [10] incorporates propagation-delay-aware long-range dependency modeling. Models such as STID [11] and STAEformer [12] further show that node identity, temporal embeddings, and adaptive spatio-temporal representations can provide effective forecasting information. These studies substantially improve traffic forecasting accuracy, but their main objective remains learning future traffic states from relatively reliable historical observations rather than systematically evaluating robustness under faulty historical sensor inputs.

In parallel with forecasting-model development, traffic data imputation and missing-aware prediction have received sustained attention. Statistical, low-rank, tensor, recurrent, graph, diffusion, adversarial, and self-attention methods recover incomplete historical observations [13,14,15,16,17,18,19,20,21,22,23,24,25,26,27,28,29,30]. Recent traffic-specific studies further combine heterogeneous vehicle-detection and dedicated short-range communication data to estimate speeds in unobserved segments [31], jointly pretrain imputation and forecasting tasks [32], and optimize missing-data recovery and traffic prediction through multi-task learning [33]. These approaches establish that downstream prediction depends on how incomplete observations are handled. Nevertheless, most imputation models primarily minimize historical reconstruction error, whereas the forecasting task is determined by whether a transformed history reduces future prediction error; the two objectives need not coincide.

Robustness research has also moved beyond conventional missing-value imputation. Uncertainty-aware traffic prediction models quantify prediction reliability at locations with incomplete histories [34], sparse adversarial training improves robustness to missing and noisy traffic inputs [35], and real-data anomaly studies identify sensor failures or irregular traffic conditions [36,37]. Related traffic-system robustness research has also considered historical-information compensation when cyber-attacks corrupt or remove connected-vehicle information [38]. However, these studies optimize different targets: uncertainty modeling estimates predictive confidence, anomaly detection identifies abnormal observations, cyber-resilient control seeks traffic-flow stability, and adversarial training hardens a forecasting backbone. They do not directly establish whether an explicit pre-forecasting repair channel can improve future speed prediction across both unavailable observations and finite-valued faults.

Motivated by this gap, this paper studies traffic speed forecasting under the controlled degradation of historical observations. Given corrupted historical speed inputs, the model predicts clean future speed targets. The runtime-visible observation mask identifies unavailable values, whereas the controlled fault-location mask is retained only by the experimental generator for offline auditing, diagnostic analysis, and controlled-oracle imputation comparisons. It is not used by the final SRAF-ID for training or inference. The generator provides a reproducible window-level stress protocol for isolating how degraded histories affect future prediction. The primary experiment uses matched train/test perturbations, while additional frozen-checkpoint tests probe unseen severities and localized per-window faults; field-recorded and event-continuous failures remain external-validation settings.

This paper proposes SRAF-ID, a prediction-oriented pre-forecasting speed-channel repair framework. Its principal methodological contribution is the information-boundary-aware integration of same-sensor temporal repair, mask-aware graph-neighborhood repair over the supplied adjacency, and adaptive two-way fusion before an identity-enhanced forecasting backbone. The design selectively transforms the sensor-measurement channel, while node identity and periodic temporal context bypass the repair front-end. Because the complete pipeline is optimized solely against clean future targets, it learns prediction-useful repair without a separate historical-reconstruction objective or fault-location supervision.

The main contributions are as follows. First, we formulate traffic speed forecasting under unavailable and finite-valued degraded histories and enforce a strict information boundary between the runtime-visible observation mask and an offline fault record used only for controlled evaluation. Second, we introduce a prediction-oriented speed-channel repair front-end that adaptively combines same-sensor temporal evidence with mask-aware graph-neighborhood evidence. Third, we preserve node-identity and temporal-context pathways while training the complete repair-and-forecasting pipeline using future forecasting loss only, eliminating fault-location supervision from the final model. Fourth, we provide a raw-time-disjoint evaluation on two public sensor networks, six matched window-level stress conditions, ten seeds, a no-repair baseline comparison and an auxiliary-loss ablation, a forecast-only architecture comparison, imputation and horizon analyses, synchronized latency measurements, and checkpoint-only tests of unseen severities and localized faults.

The remainder of this paper is organized as follows. Section 2 reviews traffic forecasting, imputation, missing-aware prediction, robust forecasting, and anomaly detection. Section 3 defines the controlled fault setting and presents SRAF-ID. Section 4 reports matched robustness, fault-wise, imputation, horizon, ablation, auxiliary-loss, distribution-shift, and latency results. Section 5 and Section 6 discuss the findings, limitations, and conclusions.

## 2. Related Work

Short-term traffic forecasting has evolved from statistical and conventional machine-learning models to deep spatio-temporal architectures. DCRNN [5], STGCN [6], Graph WaveNet [7], GMAN [8], AGCRN [9], and PDFormer [10] model diffusion, graph convolution, adaptive adjacency, attention, node-adaptive parameters, and propagation delay. STID [11] and STAEformer [12] further demonstrate the value of node identity, temporal embeddings, and adaptive spatio-temporal representation, while recent Sensors studies investigate dynamic sparse graph convolution, multi-graph gated convolution, and multiscale recurrent representations [39,40,41]. These methods principally optimize forecasting accuracy under cleaned or routinely preprocessed historical inputs; robustness to systematic degradation of the historical speed channel is usually not their primary design objective.

Traffic-data recovery research addresses missing observations through temporal neighbors, spatial neighbors, low-rank structures, tensor completion, recurrent networks, graph neural networks, diffusion models, adversarial models, and probabilistic latent variables [13,14,15,16,17,18,19,20,23,25,26,27,28,29,30]. Traffic-specific multimodal estimation can combine vehicle-detection systems and dedicated short-range communication data to estimate unobserved road segments [31]. Multi-task approaches jointly learn imputation and forecasting [32,33], and imputation-then-forecasting pipelines explicitly reconstruct the input before prediction. Their strength is direct historical recovery, but reconstruction metrics do not necessarily identify the transformed history that minimizes future forecasting error.

Missing-aware and robust forecasting methods address related but distinct problems. LSTM and tensor-completion-assisted predictors incorporate missing histories [33,42,43,44], uncertainty-aware forecasting extends prediction to locations without historical records and quantifies uncertainty [34], and SAFER-predictor uses sparse adversarial training for missing and noisy data [35]. Beyond forecasting, Zhai et al. [38] compensate cyber-attack-induced missing traffic information with historical data and a self-stabilizing control scheme, but their objective is macroscopic connected-vehicle flow stability rather than sensor-level speed prediction. Prediction-residual and clustering methods identify abnormal traffic observations or sensor failures from operational streams [36,37], but detection is not equivalent to repairing the input for downstream forecasting. Taken together, the recent literature spans reconstruction or compensation, uncertainty estimation, anomaly detection, and robust predictor training; these objectives are complementary but not interchangeable.

SRAF-ID is positioned between standalone imputation and robust forecasting. It inserts a modular speed-channel repair front-end before a common identity-enhanced multilayer perceptron (ID-MLP) backbone and learns its behavior solely from downstream forecasting error, without requiring an online unknown-fault detector or the controlled fault-location mask. The specific gap addressed here is the absence of a unified, prediction-oriented transformation that (i) handles explicit unavailability and valid-looking noise, drift, and stuck readings within one protocol; (ii) adaptively combines same-sensor temporal evidence with a mask-aware graph-neighborhood candidate over the supplied adjacency; (iii) preserves deterministic identity and periodic context outside the repair path; and (iv) is evaluated through matched-backbone, seed-paired stress tests. The resulting scope centers on robust downstream forecasting, while standalone anomaly detection and generic historical-imputation ranking remain complementary tasks.

## 3. Materials and Methods

### 3.1. Data Structure and Problem Definition

This section defines the road-sensor network, faulty historical inputs, and clean future prediction targets. The traffic sensor network is represented as a weighted graph:

(1) $$𝒢=\left(𝒱,E,A\right),\;\;\left|𝒱\right|=N,\;\;A\in R^{N\times N}.$$

where 𝒱 is the set of sensor nodes, E is the supplied set of spatial relations, |𝒱| = N, and A ∈ ℝ^N × N^ is the weighted adjacency matrix. The DCRNN-compatible adjacency pickle supplied for each dataset is loaded without constructing new off-diagonal edges or applying an additional distance threshold. Although the saved matrices contain unit diagonal entries, the implemented graph-neighborhood operator removes those self-loops. At each sample and history step, weights of neighbors with Ω^obs^ = 0 are removed and the remaining visible-neighbor weights are renormalized. M^fault^ is never used to alter the graph weights.

Let B denote batch size, L the historical window length, and H the prediction horizon. The clean historical speed tensor X and clean future target Y are defined in Equation (2). For notational compactness, Equation (2) suppresses the singleton speed-channel dimension used in the implementation, where the tensors are stored as [B, L, N, 1] and [B, H, N, 1].

(2) $$X\in R^{B\times L\times N},\;\;Y\in R^{B\times H\times N}.$$

The controlled generator produces a corrupted history X^c^ together with two masks. Ω^obs^ is the model-visible observation-availability mask, while M^fault^ records positions modified by the controlled protocol. Missing and outage positions have Ω^obs^ = 0; finite-valued noise, drift, and stuck readings generally retain Ω^obs^ = 1. M^fault^ is excluded from the revised model’s training and inference mappings and is used only for offline protocol auditing, diagnostics, and controlled-oracle comparisons.

(3) $$\Omega_{b,i,n}^{obs}=\{\begin{array}{ll} 1, & X_{b,i,n}^{c}\;\mathrm{is}\;\mathrm{available}\;\mathrm{and}\;\mathrm{finite},\\ 0, & X_{b,i,n}^{c}\;\mathrm{is}\;\mathrm{missing}\;\mathrm{or}\;\mathrm{unavailable}. \end{array}\;M_{b,i,n}^{fault}=\{\begin{array}{ll} 1, & \left(b,i,n\right)\;\mathrm{is}\;\mathrm{corrupted}\;\mathrm{by}\;\mathrm{the}\;\mathrm{controlled}\;\mathrm{fault}\;\mathrm{generator},\\ 0, & \mathrm{otherwise}. \end{array}$$

Missing and outage entries are first represented as NaN and then replaced by the fallback scalar x_fill_ = 0 in standardized model space to preserve tensor shape. Equation (4) defines the zero-filled input $\tilde{X}$. The placeholder is accompanied by Ω^obs^ and is therefore not interpreted as a valid zero speed. Finite-valued faults remain numerically present in $\tilde{X}$ and must be handled through local variation and candidate-disagreement features rather than through an availability flag.

(4) $${\tilde{X}}_{b,i,n}=\Omega_{b,i,n}^{obs}X_{b,i,n}^{c}+\left(1-\Omega_{b,i,n}^{obs}\right)x_{fill}.$$

In addition to speed, the model uses deterministic contextual features: node identity Z_node_, time-of-day Z_tod_, and day-of-week Z_dow_. These variables are generated from sensor indices and timestamps and remain unchanged under the fault protocol. Equation (5) defines the forecasting mapping from the zero-filled history, observation mask, adjacency, and contextual features to the clean future prediction Ŷ.

(5) $$\hat{Y}=f_{\theta }\left(\tilde{X},\Omega^{obs},A,Z^{node},Z^{tod},Z^{dow}\right).$$

Faults are applied only to the historical speed channel, whereas the future target Y remains clean. The evaluation metrics therefore measure whether the explicit repair front-end improves future speed prediction under unreliable histories. Because M^fault^ is absent from Equation (5), the revised SRAF-ID does not assume labelled fault locations during either training or inference.

For clarity, X^c^ denotes corrupted historical speed, $\tilde{X}$ the zero-filled model input, X^temp^ and X^sp^ the temporal and graph-neighborhood candidates, X^fuse^ their adaptive combination, X^rep^ the final repaired history, Y the clean future target, and Ŷ the multi-step prediction. Ω^obs^ is model-visible; M^fault^ is an offline experimental record.

### 3.2. Faulty Observation Modeling

Given a clean historical speed tensor X, the controlled fault operator C_τ_ returns the corrupted history X^c^, the model-visible observation mask Ω^obs^, and the offline fault record M^fault^. Here, τ denotes the fault type and θ_τ_ denotes its parameter set; the fault-specific parameters are defined separately in Equations (7)–(11).

(6) $$\left(X^{c},\Omega^{\mathrm{obs}},M^{\mathrm{fault}})=C_{\tau }(X;\theta_{\tau }\right)$$

The protocol abstracts abnormal operation as sensor-observation degradation and does not introduce accident or disaster labels. Perturbations are generated independently for each already-formed historical window, while the future target remains clean. Consequently, overlapping windows need not assign the same fault state to the same underlying raw observation. M^fault^ is retained for reproducible fault accounting, candidate diagnostics, seed-level statistics, and controlled-oracle imputation, but it is not an input to SRAF-ID and does not contribute to its final training loss.

Random missing faults simulate packet loss or local sensor data absence. For random missing with rate *p*, the speed-channel mask is sampled at the sensor-time position level:

(7) $$M_{b,i,n}^{fault}∼Bernoulli\left(p\right),\;\Omega_{b,i,n}^{obs}=1-M_{b,i,n}^{fault}.\;X_{b,i,n}^{c}=\{\begin{array}{ll} NaN, & M_{b,i,n}^{fault}=1,\\ X_{b,i,n}, & M_{b,i,n}^{fault}=0. \end{array}$$

For random missing faults, faults occur at sensor-time positions. Selected historical speed positions are written as NaN and then zero-filled before model input; these positions satisfy Ωobs = 0 and Mfault = 1. This paper reports RM20 and RM40, corresponding to *p* = 0.20 and *p* = 0.40, respectively.

Continuous outage perturbations remove consecutive observations within each input window. Let S_b_ be the set of sensors selected for window b, and let s_b_ be the within-window outage start index. A continuous-outage perturbation with requested length l is represented as

(8) $$\left|S_{b}\right|=max\{1,round\left(0.10N\right)\}\;l\prime =min\left(24,L\right)\;s_{b}∼Uniform\{0,\ldots ,L-l\prime \}\;M_{b,i,n}^{fault}=1\;\mathrm{if}\;n\in S_{b},\;i\in \left[s_{b},s_{b}+l\prime -1\right]$$

For continuous outage, perturbations occur as sensor-level consecutive blocks within a window. For each sample, max(1, round(0.10N)) sensors are sampled without replacement and *s_b_* is sampled from {0, …, L − l′}, where l′ = min(24, L). Since L = 12, the requested 24-step block covers the complete visible history; this condition is reported as CO-full. Sensor selections are resampled by window and are not intended to reproduce one continuous physical outage across overlapping windows.

Additive noise, linear drift, and stuck readings simulate sensor observations that remain numerically present but semantically unreliable. The three faults are defined as

(9) $$X_{b,i,n}^{c}=X_{b,i,n}+\epsilon_{b,i,n},\;\epsilon_{b,i,n}∼N\left(0,\sigma^{2}\right),\;\sigma =0.50\;\sigma_{train}.$$

(10) $$X_{b,i,n}^{c}=X_{b,i,n}+\alpha_{i}\cdot 0.50\;\sigma_{train},\;\alpha_{i}=\frac{i}{L-1},\;i=0,\ldots ,L-1.$$

(11) $$\left|S_{b}\right|=max\{1,round\left(0.50N\right)\},\;l_{stuck}=round\left(\left(L-1\right)\times 0.50\right)=6\;\left(L=12\right).\;X_{b,i,n}^{c}=X_{b,s_{b}-1,n},\;i=s_{b},\ldots ,s_{b}+l_{stuck}-1,\;n\in S_{b},\;s_{b}∼Uniform\{1,\ldots ,L-l_{stuck}\}.$$

For finite-valued perturbations, corrupted values remain finite and the observation-availability mask remains one. GN-high adds independent Gaussian noise with a standard deviation equal to 0.50 times the training-set standard deviation to every sensor-time entry in a corrupted window. LD-high adds the same positive zero-to-0.50 standard-deviation ramp to every sensor in each window, with the ramp restarting at the beginning of the next window. SV-high resamples max(1, round(0.50N)) sensors per window and replaces a six-step segment with its immediately preceding value. Together with missingness and outage, these conditions deliberately span availability failures, network-wide finite-valued perturbations, and window-local persistent faults within one reproducible protocol.

#### Operational Interpretation and Severity Rationale

**Figure 1:**
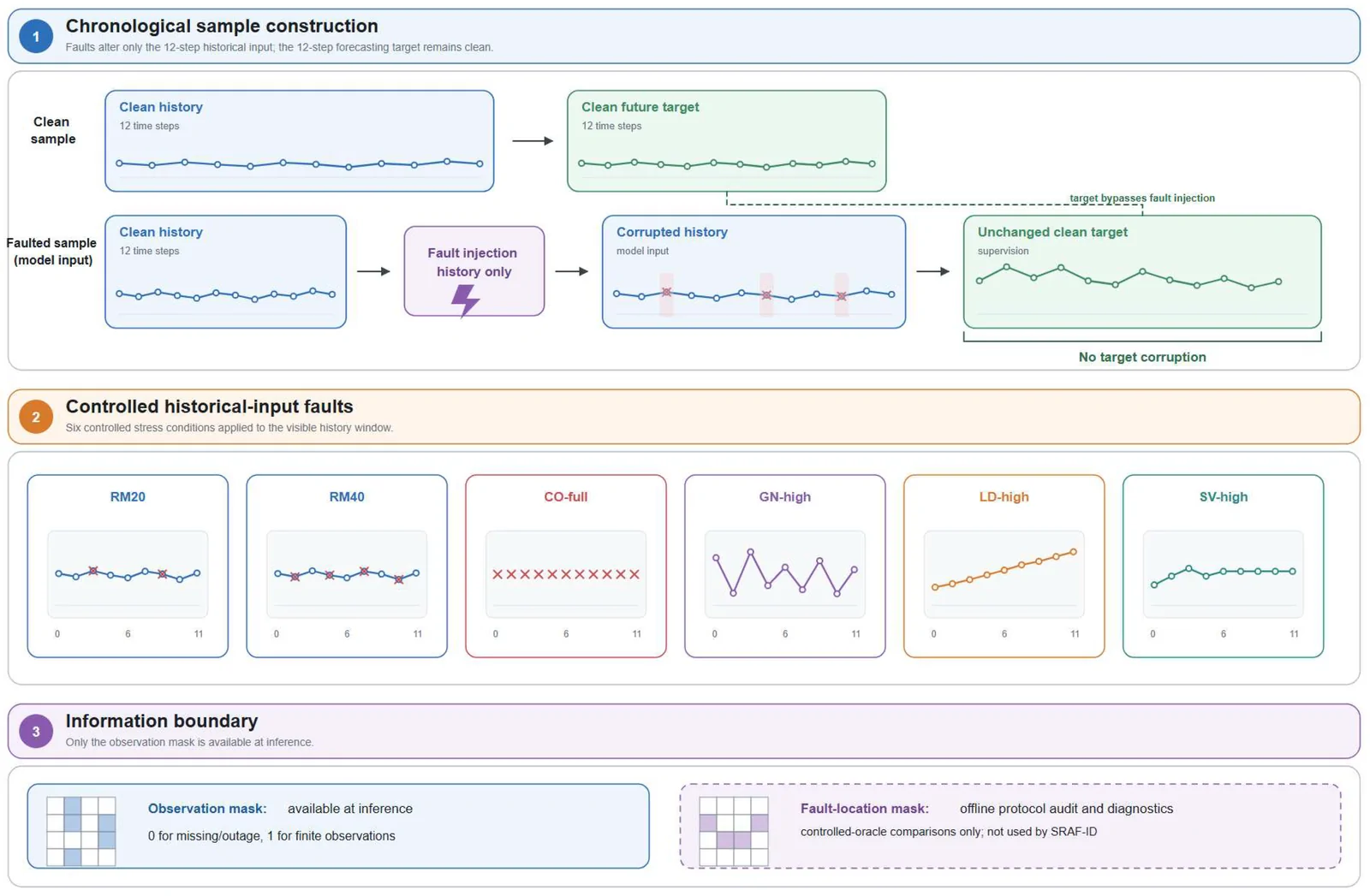
Window-level controlled faulty-observation protocol. Perturbations are restricted to the historical input, and the future target remains clean. RM20, RM40, CO-full, GN-high, LD-high, and SV-high are matched synthetic stress conditions. Only the observation mask is available to SRAF-ID; the fault-location record is retained offline for auditing, diagnostics, and controlled-oracle comparisons. Numbered bullets denote protocol stages, and colors distinguish stages and fault types; neither encodes quantitative magnitude.

The perturbation mechanisms are operationally motivated by documented detector failures, missing transmissions, noisy measurements, persistent constant readings, and other abnormal sensor sequences [2,21,22,36,37]. The selected numerical severities define reproducible stress levels rather than field-frequency estimates: RM20 and RM40 impose 20% and 40% position loss; CO-full removes the full 12-step history for 10% of sensors per window; GN-high and LD-high use 0.50 times the training-set standard deviation; and SV-high affects 50% of sensors for six steps. Training and formal testing use the same family of window-level perturbations. This matched design supports auditable cross-model comparisons within a specified stress envelope. To probe two additional boundaries without retraining, Section 4.9 evaluates lower and higher Gaussian-noise and linear-drift severities, 5%-sensor-localized noise and drift, and one-sensor outage and stuck-value conditions. These additions remain window-level tests and do not estimate field prevalence, preserve event continuity, or introduce unseen fault families. Figure 1 summarizes the window-level controlled fault protocol and the information boundary between the model-visible observation mask and the offline fault record.

### 3.3. Overall Architecture of SRAF-ID

SRAF-ID constructs a repaired speed input before the forecasting backbone while leaving node identity, time-of-day, and day-of-week features unchanged. Here, “sensor-reliability-aware” denotes the use of observation availability, local temporal consistency, and temporal-spatial candidate disagreement to adapt repair weights; it does not denote an explicit sensor-health classifier or online unknown-fault detector. The suffix “ID” denotes the identity-preserved design in which deterministic node and temporal features bypass the repair channel. The revised framework is trained end to end with future forecasting loss only and does not require fault-location supervision.

(12) $$X^{\mathrm{temp}}=\phi_{\mathrm{temp}}(\overset{\sim }{X},\Omega^{\mathrm{obs}}),X^{\mathrm{sp}}=\phi_{\mathrm{sp}}(\overset{\sim }{X},\Omega^{\mathrm{obs}},A).$$

The architecture follows a selective speed-channel repair principle. Historical speed is a sensor measurement and may be affected by device, communication, or calibration errors; node identity, time-of-day, and day-of-week information are determined by indices and timestamps and are deterministic contextual features. SRAF-ID therefore repairs only historical speed observations while passing identity and temporal features to the forecasting backbone through a contextual bypass.

**Figure 2:**
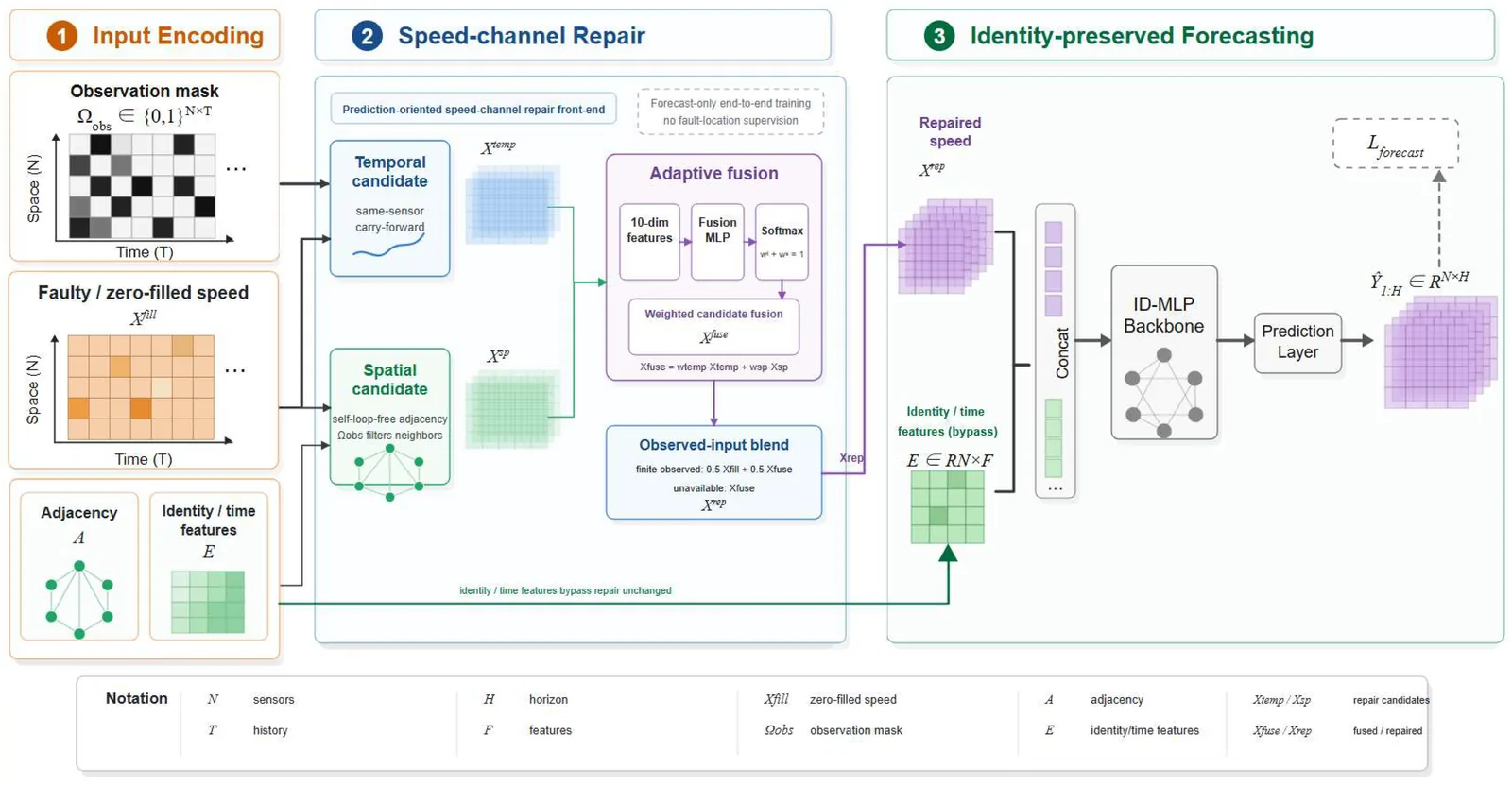
Overall architecture of the revised SRAF-ID. The historical speed channel is processed by temporal and mask-aware graph-neighborhood candidates, learned two-way softmax fusion, and the observed-input blend. The graph branch uses Ω^obs^ to exclude unavailable neighbors but never uses M^fault^. Deterministic node-identity and temporal-context features bypass repair. The complete forward path is optimized end to end using future forecasting loss, without fault-location supervision. Numbered bullets indicate processing stages, arrows indicate data flow, and colors distinguish functional modules without encoding quantitative magnitude.

Compared with training a predictor directly on corrupted speeds, SRAF-ID explicitly constructs a repaired speed channel before forecasting. As shown in Figure 2, the zero-filled speed and observation mask produce a same-sensor temporal candidate. The mask-aware graph-neighborhood branch removes adjacency self-loops, filters neighbors with Ω^obs^ = 0, and renormalizes the remaining supplied edge weights; it does not use M^fault^. A fusion MLP assigns two-way softmax weights, after which the observed-input blend produces X^rep^. Node identity, time-of-day, and day-of-week features bypass the repair front-end and are concatenated before the ID-MLP backbone. Forecasting loss supervises the complete repair-and-forecasting pipeline end to end.

### 3.4. Speed-Channel Repair Module

The repair front-end consists of a same-sensor temporal candidate, a mask-aware graph-neighborhood candidate based on the supplied adjacency, and two-way softmax fusion over local repair-related features. It does not introduce a time-of-day profile candidate or an additional reliability gate in the final architecture. Rather than minimizing an independent reconstruction objective, the front-end is optimized solely according to its contribution to future forecasting accuracy while preserving identity and temporal-context information in the backbone.

#### 3.4.1. Temporal Repair Candidate

The temporal candidate X^temp^ adopts a carry-forward recurrence. If the current position is a finite observation, $\tilde{X}$ is retained. If the current position is unavailable, the temporal candidate from the previous time step of the same sensor is used. If the first time step is unavailable, the fallback value x_fill_ is used. This candidate does not use attention-based temporal aggregation; instead, it recursively preserves the most recent finite observation of the same sensor, mainly for random missing and short outage scenarios.

(13) $$X_{b,i,n}^{\mathrm{temp}}=\{\begin{array}{cc} {\overset{\sim }{X}}_{b,i,n} & \Omega_{b,i,n}^{\mathrm{obs}}=1,\\ X_{b,i-1,n}^{\mathrm{temp}} & \Omega_{b,i,n}^{\mathrm{obs}}=0,i>0,\\ x_{\mathrm{fill}} & \Omega_{b,i,n}^{\mathrm{obs}}=0,i=0. \end{array}$$

When a sensor is continuously unavailable throughout the input window or when a stuck reading persists for a long segment, effective temporal evidence within the same sensor is reduced. In such cases, the temporal candidate must be combined with the spatial candidate to mitigate error propagation caused by insufficient temporal evidence.

#### 3.4.2. Spatial Repair Candidate

The mask-aware graph-neighborhood candidate X^sp^ aggregates zero-filled same-time-step speeds using the supplied weighted adjacency matrix. The implemented operator removes diagonal self-loops, multiplies each remaining neighbor weight by the model-visible availability Ω^obs^, and renormalizes the visible-neighbor weights for each sample and history step. If no weighted visible neighbor remains, it falls back to X^temp^. M^fault^ is not used.

(14) $$A_{b,i,n,j}^{\mathrm{eff}}=A_{n,j}\Omega_{b,i,j}^{\mathrm{obs}}(1-\delta_{n,j}).$$

(15) $$X_{b,i,n}^{\mathrm{sp}}=\{\begin{array}{cc} \frac{\sum_{j=1}^{N}A_{b,i,n,j}^{\mathrm{eff}}{\overset{\sim }{X}}_{b,i,j}}{\sum_{j=1}^{N}A_{b,i,n,j}^{\mathrm{eff}}} & \sum_{j=1}^{N}A_{b,i,n,j}^{\mathrm{eff}}>\varepsilon ,\\ X_{b,i,n}^{\mathrm{temp}} & \mathrm{otherwise}. \end{array}$$

Equation (14) defines the effective graph-neighborhood weights after self-loop removal and Ωobs filtering, where δ_n,j_ is the Kronecker delta. Equation (15) applies sample- and time-specific visible-neighbor renormalization, with numerical tolerance ε = 10^−6^ and Xtemp as the zero-denominator fallback. This uses only runtime-visible availability and the supplied off-diagonal adjacency; it does not use Mfault or any finite-valued-fault label. Equations (16)–(19) then define the softmax fusion.

The spatial candidate can provide adjacent-sensor evidence for continuous outages, isolated noise, and stuck readings, but its reliability depends on neighboring sensor states and local traffic heterogeneity. When adjacent road segments differ in direction, ramp influence, or congestion propagation delay, the spatial candidate may deviate from the true state of the target sensor. SRAF-ID therefore learns fusion weights between temporal and spatial candidates.

#### 3.4.3. Adaptive Temporal-Spatial Fusion

The fusion module generates two-way softmax weights for the temporal and graph-neighborhood candidates. Its ten input features are the zero-filled speed, observation mask, absolute first-order difference, local variance, speed magnitude, absolute second-order difference, two filled-speed-to-candidate gaps, temporal-spatial candidate gap, and a stuck-duration estimate. Δ$\tilde{X}$ is the backward first difference with zero at i = 0; Δ^2^$\tilde{X}$ is the second backward difference with zeros at i = 0 and 1. Var_local_ uses a three-step centered window with replicated boundary values. The stuck feature is an approximate normalized run-length proxy: from the second history step onward, it increments when the current position is observed and the current and previous zero-filled values differ by less than 10^−6^, resets otherwise, and is divided by max(1, L − 1). Because the observation mask remains one for finite-valued faults, fusion is driven by local consistency and candidate disagreement. Neither the features nor the final objective uses the controlled fault-location mask.

For position (i,n), the local repair-related features are defined as

(16) $$\begin{array}{c} q_{b,i,n}=[{\overset{\sim }{X}}_{b,i,n},\Omega_{b,i,n}^{\mathrm{obs}},|\Delta {\overset{\sim }{X}}_{b,i,n}|,{Var}_{\mathrm{local}}(\overset{∼}{X}{)}_{b,i,n},|{\overset{\sim }{X}}_{b,i,n}|,\\ |\Delta^{2}{\overset{\sim }{X}}_{b,i,n}|,|{\overset{\sim }{X}}_{b,i,n}-X_{b,i,n}^{\mathrm{sp}}|,|{\overset{\sim }{X}}_{b,i,n}-X_{b,i,n}^{\mathrm{temp}}|,|X_{b,i,n}^{\mathrm{temp}}-X_{b,i,n}^{\mathrm{sp}}|,r_{b,i,n}^{\mathrm{stuck}}{]}^{T}. \end{array}$$

The fusion MLP outputs a two-dimensional score vector s_i,n_; a softmax operation then yields the temporal-candidate weight and the spatial-candidate weight:

(17) $$\left[s_{b,i,n}^{temp},s_{b,i,n}^{sp}\right]={MLP}_{fusion}\left(q_{b,i,n}\right),\;\left[w_{b,i,n}^{temp},w_{b,i,n}^{sp}\right]=softmax\left(\left[s_{b,i,n}^{temp},s_{b,i,n}^{sp}\right]\right)$$

The two weights are non-negative and normalized:

(18) $$w_{b,i,n}^{temp}\geq 0,\;w_{b,i,n}^{sp}\geq 0,\;w_{b,i,n}^{temp}+w_{b,i,n}^{sp}=1\;X_{b,i,n}^{fuse}=w_{b,i,n}^{temp}X_{b,i,n}^{temp}+w_{b,i,n}^{sp}X_{b,i,n}^{sp}$$

The candidate fusion and the final repaired speed after the observed-input blend are defined as

(19) $$X_{b,i,n}^{rep}=\Omega_{b,i,n}^{obs}\left(0.5{\tilde{X}}_{b,i,n}+0.5X_{b,i,n}^{fuse}\right)+\left(1-\Omega_{b,i,n}^{obs}\right)X_{b,i,n}^{fuse}$$

**Figure 3:**
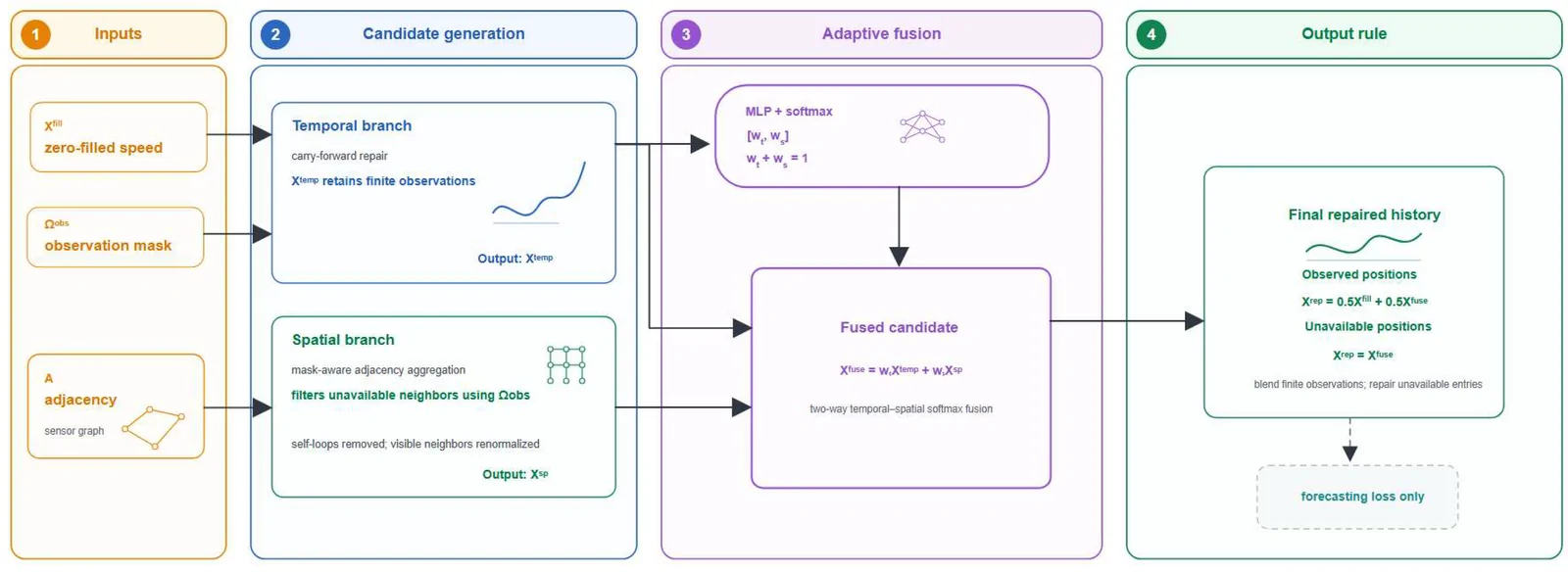
Speed-channel repair module. The temporal branch carries forward same-sensor observations. The graph-neighborhood branch removes self-loops, filters unavailable neighbors using Ω^obs^, and renormalizes the remaining supplied adjacency weights without using M^fault^. Ten local features drive a two-way softmax over the two candidates; the observed-input blend then produces the final repaired history. Numbered bullets indicate processing stages, arrows indicate data flow, and colors distinguish functional modules.

The two-way normalization makes the temporal and mask-aware graph-neighborhood candidates comparable within the same weight space. When same-sensor temporal evidence is more reliable, the model can assign a larger temporal weight; when local temporal information is degraded, it can rely more on visible neighbors. For finite observed positions, a fixed observed-input blend coefficient of 0.5 combines the zero-filled finite speed with the fused candidate, whereas unavailable positions directly use the fused candidate. The coefficient 0.5 was fixed a priori as a symmetric interpolation; it was not selected through test-set optimization and is not claimed to be universally optimal. Figure 3 details the two candidate branches, the ten fusion features, the two-way softmax, and the final output rule. Supplementary Figures S1 and S2 provide descriptive diagnostics, and Supplementary Figure S3 audits the raw-time-disjoint split.

### 3.5. Identity-Preserved Forecasting Backbone

After speed-channel repair, SRAF-ID feeds X^rep^ and deterministic contextual features into the ID-MLP forecasting backbone. The backbone uses node identity and temporal identity to represent sensor-specific differences and periodic patterns [11]. Let E^node^, E^tod^, and E^dow^ denote the node-identity, time-of-day, and day-of-week embeddings, respectively. The forecasting input is

(20) $$U=\left[X^{rep},E^{node},E^{tod},E^{dow}\right]$$

The forecasting backbone learns the mapping from U to the future H-step speed sequence:

(21) $$\hat{Y}=f_{\theta }^{ID\text{-}MLP}\left(U\right).$$

The same-backbone baseline ID-MLP-CA uses the same forecasting backbone and the same clean future target, but it does not include an explicit speed repair module. Its input is

(22) $$U_{CA}=\left[\tilde{X},E_{node},E_{tod},E_{dow}\right],\;{\hat{Y}}_{CA}=f_{\theta }^{ID\text{-}MLP}\left(U_{CA}\right).$$

The key difference between SRAF-ID and ID-MLP-CA is whether an explicit repaired speed channel is constructed before the shared forecasting backbone. ID-MLP-CA directly feeds corrupted speed and deterministic context into the ID-MLP backbone. SRAF-ID first generates temporal and spatial repair candidates, adaptively combines them, and then supplies the repaired speed with the same contextual features. Both models are trained against the same clean future target using the same forecasting objective and share the forecasting backbone and fault distribution. However, this comparison is not a strict repair-only ablation because SRAF-ID additionally receives the runtime-visible observation mask, whereas ID-MLP-CA does not. The measured difference therefore reflects the complete SRAF-ID input-and-repair design rather than the isolated contribution of the repair transformation alone.

### 3.6. Training Objective

The revised SRAF-ID is trained using the future speed forecasting loss only. The objective measures mean absolute error between the multi-step prediction Ŷ and clean future target Y and directly optimizes downstream forecasting performance.

For a batch of B samples, H prediction steps, and N sensors, Equation (23) defines the final objective. The temporal candidate, spatial candidate, adaptive fusion, and forecasting backbone are fully differentiable, so forecasting gradients propagate through the complete repair front-end.

The controlled fault-location mask is not used in Equation (23). Consequently, the final SRAF-ID requires neither labelled fault locations nor a fault-location-based auxiliary target during final training or inference.

(23) $$L_{SRAF\text{-}ID}=L_{forecast}=\frac{1}{BHN}\sum_{b=1}^{B}\sum_{h=1}^{H}\sum_{n=1}^{N}\left|{\hat{Y}}_{b,h,n}-Y_{b,h,n}\right|.$$

An auxiliary historical repair loss with weight λ_repair_ = 0.05 was included as a heuristic weak-supervision term in the original submission. Specifically, this auxiliary loss was the sum of the absolute repaired-versus-clean history error at controlled fault positions, divided by the larger of one and the number of such positions. The historical objective added λ_repair_ times this auxiliary loss to the future forecasting loss. Under the corrected raw-time-disjoint split, a ten-seed paired comparison of λ_repair_ = 0 and 0.05 shows no stable forecasting benefit from the auxiliary term. The final model therefore uses λ_repair_ = 0 and forecast-only training, so neither the auxiliary loss nor the offline fault mask contributes to final parameter updates. ID-MLP-CA uses the same future forecasting objective.

## 4. Results

### 4.1. Datasets

SRAF-ID is evaluated on two public traffic speed datasets, METR-LA and PEMS-BAY. Both datasets are collected from fixed road sensors and are widely used benchmarks for traffic speed forecasting and incomplete traffic data modeling [45]. METR-LA contains 207 sensors, and PEMS-BAY contains 325 sensors. All experiments use a historical window of L = 12 and a prediction horizon of H = 12, forecasting the next 60 min of speed observations from the previous 60 min. Table 1 summarizes the data scale, forecasting protocol, and random seed setting used in the experiments.

### 4.2. Baseline Models

To evaluate the effect of speed-channel repair on downstream prediction, the experiments use a unified ID-MLP forecasting backbone. ID-MLP denotes the base forecaster, while ID-MLP-CA is a corruption-aware same-backbone baseline trained on degraded inputs without an explicit repair front-end. The final SRAF-ID adds the temporal-spatial speed-repair module before the same backbone and is trained using forecasting loss only. KNN+ID-MLP, PPCA-lite+ID-MLP, and PyPOTS-SAITS+ID-MLP represent imputation-then-forecasting pipelines. The primary final-objective architecture comparison retrains Temporal-only with the same λ_repair_ = 0 objective as SRAF-ID; the earlier five-variant matrix with a common λ_repair_ = 0.05 objective is retained as secondary component evidence in Supplementary Table S10.

### 4.3. Experimental Settings and Evaluation Metrics

The formal experiments use the controlled faulty-observation protocol defined in Section 3.2. Table 2 summarizes the fault abbreviations, key parameters, and model-visible boundaries used in the main text. All faults are applied only to the historical speed input; node identity, time-of-day, day-of-week information, and the future target Y remain clean. This setting isolates the effect of historical speed observation degradation on forecasting performance.

Relative gain measures the MAE reduction of SRAF-ID over a comparison model under the same dataset and fault setting and is defined as

$$Gain=\frac{{MAE}_{baseline}-{MAE}_{model}}{{MAE}_{baseline}}\times 100\%.$$

All formal results use ten random seeds (42–51) and complete raw-time-disjoint training, validation, and held-out test splits. For each dataset, model, and seed, one checkpoint is trained using a deterministic rotation of five window-level perturbations: CO-full, RM40, GN-high, LD-high, and SV-high. For both SRAF-ID and ID-MLP-CA, checkpoint selection uses the validation-only score S_val_ = 0.5 MAE_clean_ + 0.5 mean (MAE_RM40_, MAE_GN-high_, MAE_SV-high_), where the three fixed corrupted validation sets are generated separately for each seed, and all four terms are computed in standardized model space. The held-out test split is never used for training, early stopping, checkpoint choice, or hyperparameter selection. Matched ID-MLP-CA and final SRAF-ID runs share raw speed windows, clean targets, deterministic context, split boundaries, perturbation rotation, data order, validation protocol, and seeds. SRAF-ID additionally receives Ω^obs^ and its repair front-end; ID-MLP-CA receives the same zero-filled corrupted speed and context directly, without a repair module or fault-location labels.

Training uses Adam for at most 60 epochs, early-stopping patience of 10, batch size 64, learning rate 0.002, weight decay 0.0001, and gradient clipping at 5.0. MAE is the primary metric, and RMSE is reported in Supplementary Table S2. For aggregate faulty-input results, the six fault MAEs are first averaged within each seed; means, sample standard deviations (ddof = 1), and 95% t confidence intervals are then computed across the ten seed-level averages. Paired tests are interpreted as descriptive stability evidence rather than confirmatory family-wise inference.

The imputation comparisons use the same raw-time-disjoint split and held-out test targets. Each pipeline trains its own ID-MLP forecaster on the clean training histories; SAITS is fitted on the training split, whereas KNN and PPCA-lite are nonparametric test-time imputers. At evaluation, the controlled generator supplies M^fault^ to define the positions replaced by KNN, PPCA-lite, or SAITS. These are therefore controlled-oracle imputation-then-forecasting comparisons with a different information boundary from SRAF-ID and should be interpreted separately from the primary SRAF-ID versus ID-MLP-CA comparison.

The raw chronological series is split into 70%/10%/20% train/validation/test segments before any 12-step history and 12-step target windows are formed. This yields 23,967/3404/6832 windows for METR-LA and 36,458/5188/10,401 windows for PEMS-BAY. Auditing the raw timestamps used by the resulting windows gives zero train–validation, train–test, and validation–test intersections for both datasets. The test set is therefore a held-out within-dataset split rather than a separate external network.

Mean and standard deviation are computed from the raw-time-disjoint training input split only and then applied unchanged to validation and test inputs and targets. Controlled perturbations are injected after window formation in standardized space, with σ_train_ = 1 for GN-high and LD-high. MAE and RMSE are computed after inverse transformation to the original speed scale (miles per hour, mph); candidate-repair diagnostics are explicitly labelled when retained in standardized model space.

The supplied DCRNN-compatible weighted adjacency matrices are loaded without adding off-diagonal edges or applying a new distance threshold. Although the saved matrices contain unit diagonal entries, the implemented graph-neighborhood operator removes self-loops, excludes neighbors with Ω^obs^ = 0, and renormalizes the remaining visible-neighbor weights independently for each sample and history step. If no weighted visible neighbor remains, the temporal candidate is used.

The experiments were executed in Python 3.9.23 with PyTorch 2.4.1+cu121 and its CUDA 12.1 runtime on a 64-bit Microsoft Windows 11 workstation equipped with a 13th Gen Intel Core i9-13900HX CPU and an NVIDIA GeForce RTX 4060 Laptop GPU. Formal training and evaluation used CUDA. The additional distribution-shift evaluation reuses the frozen λ_repair_ = 0 SRAF-ID and ID-MLP-CA checkpoints without parameter updates. It tests global GN and LD at 0.25 and 0.75 training-set standard deviations, GN and LD localized to 5% of sensors at 0.50 standard deviations, one-sensor full-window outage, and one-sensor six-step stuck values. Local sensor sets are resampled per held-out window; therefore, these tests assess localization and severity shift, not physical event continuity or occurrence frequency.

### 4.4. Overall Robustness Results

**Figure 4:**
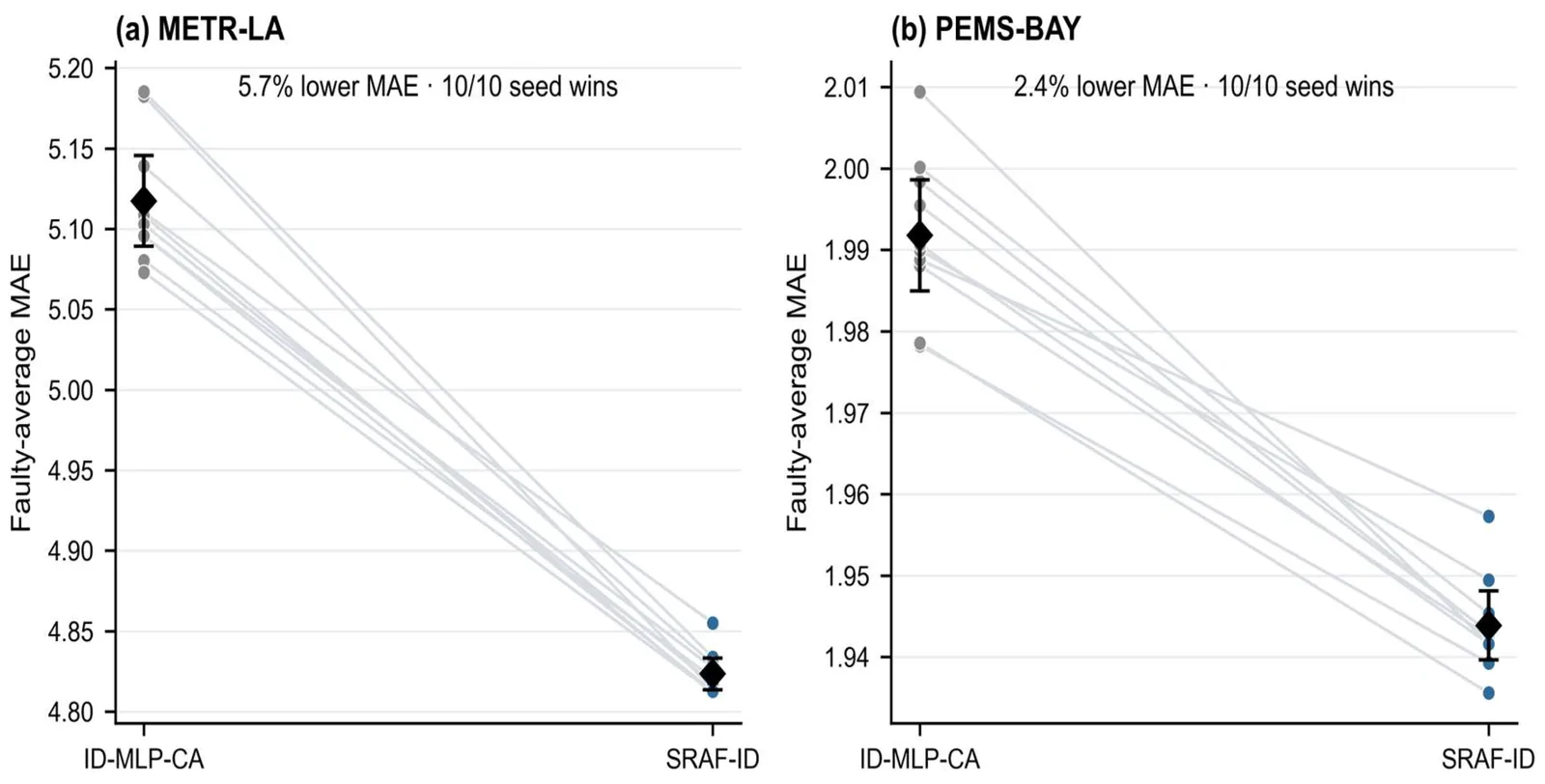
Ten-seed paired faulty-average MAE comparison under the matched protocol. Blue circles denote individual seed-level MAE values; thin gray lines connect matched seeds; black diamonds and error bars denote model means and 95% confidence intervals.

The primary comparison uses the fault-distribution-matched ID-MLP-CA baseline on the raw-time-disjoint split. Figure 4 reports faulty-average MAE, and Table 3 summarizes faulty and clean input. On METR-LA, SRAF-ID reduces faulty-average MAE from 5.1173 to 4.8234, an absolute decrease of 0.2939 and a relative reduction of 5.743%. On PEMS-BAY, MAE decreases from 1.9918 to 1.9439, an absolute reduction of 0.0479 or 2.404%. The paired 95% intervals for SRAF-ID minus ID-MLP-CA are [−0.3219, −0.2659] and [−0.0552, −0.0405], respectively, with lower faulty-average MAE in all ten paired seeds.

Under clean input, SRAF-ID obtains 4.6355 ± 0.0145 on METR-LA and 1.8026 ± 0.0037 on PEMS-BAY, compared with 4.7341 ± 0.0274 and 1.8171 ± 0.0097 for ID-MLP-CA. The relative reductions are 2.082% and 0.796%, respectively. These rows characterize nominal behavior; the primary analysis concerns controlled degradation of historical observations.

### 4.5. Fault-Type Robustness Analysis

**Figure 5:**
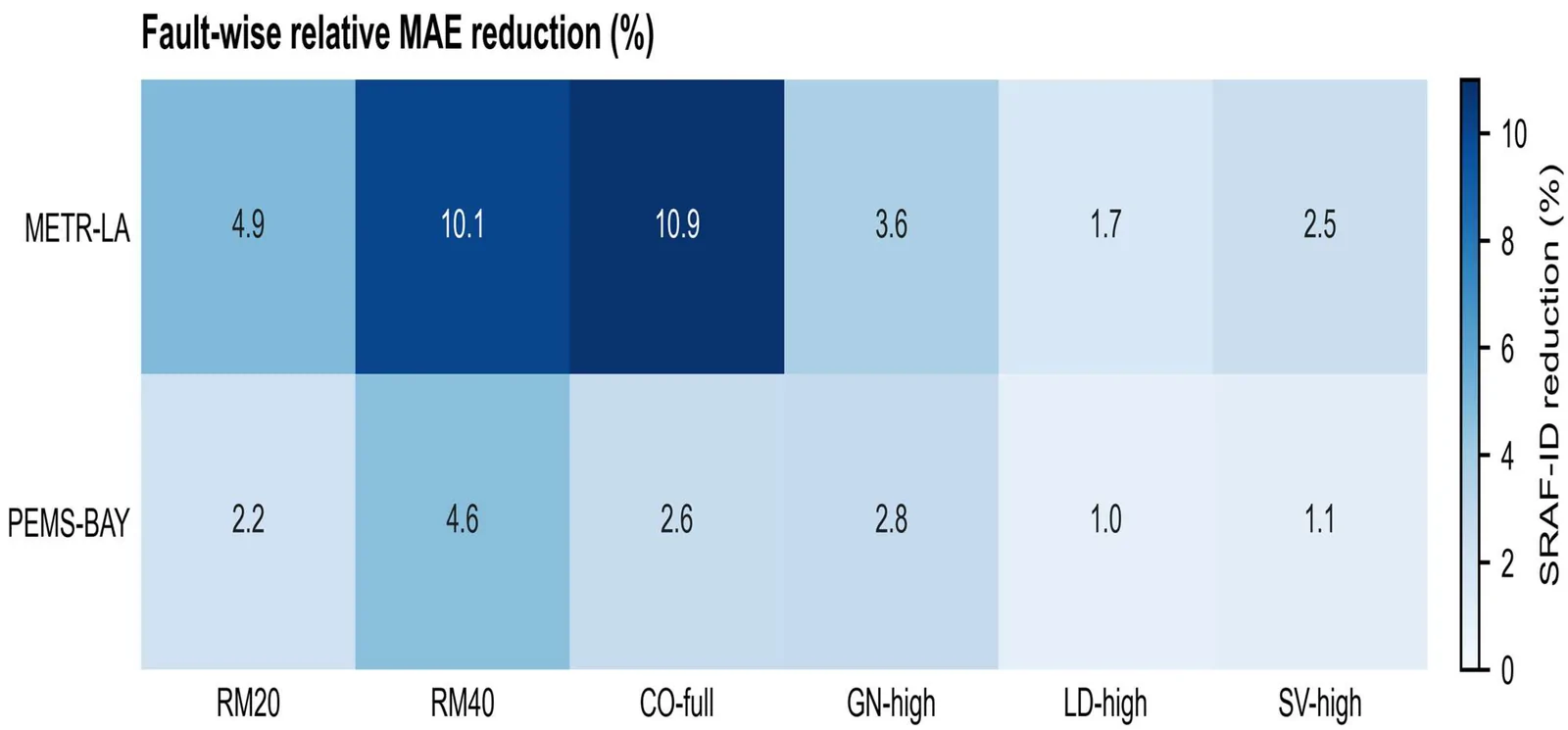
Fault-wise relative MAE reduction of SRAF-ID over ID-MLP-CA under the matched protocol. Cell values are percentages; darker shading indicates a larger reduction.

Throughout this subsection, a paired difference is defined as SRAF-ID MAE minus ID-MLP-CA MAE; negative values favor SRAF-ID. Figure 5 reports the relative MAE reduction for each dataset-fault combination, and Supplementary Table S3 provides complete seed-level statistics. Under the corrected raw-time-disjoint protocol, SRAF-ID has lower mean MAE in all 12 combinations, but the magnitude remains strongly dataset- and fault-dependent. The largest reductions occur for METR-LA CO-full (10.86%) and RM40 (10.09%); the smallest are PEMS-BAY LD-high (0.97%) and SV-high (1.09%).

The fault-wise results support bounded rather than universal robustness. SRAF-ID records 10/10 lower-MAE seeds in ten combinations and 9/10 in METR-LA LD-high and PEMS-BAY LD-high. For PEMS-BAY LD-high, the mean difference is −0.0197 with a paired 95% interval [−0.0316, −0.0078]; this small positive MAE result replaces the negative value in the earlier window-then-split analysis. MAE is still not uniformly improved across all metrics: METR-LA LD-high RMSE increases from 12.2607 to 12.3542.

Post hoc diagnostics help qualify the smaller PEMS-BAY gains without implying a forecasting failure. On the raw-time-disjoint clean test streams, mean correlation over directed nonzero off-diagonal adjacency edges is 0.7612 for METR-LA and 0.5950 for PEMS-BAY, with 0.86% and 18.24% of edges below 0.3. For PEMS-BAY LD-high, standardized candidate MAE at controlled positions is 0.2500 for the temporal candidate, 0.4655 for the graph-neighborhood candidate, 0.2684 after candidate fusion, and 0.2585 after the observed-input blend. These complete-test, ten-seed diagnostics were not used for selection and do not establish causality; they only show that graph-neighborhood evidence is less accurate than same-sensor temporal evidence for this perturbation.

Paired MAE differences are computed within each seed, with complete statistics in Supplementary Table S1. The intervals and tests characterize seed-level consistency; they are not interpreted as strict confirmatory tests under family-wise error control.

### 4.6. Imputation Comparison and Horizon-Specific Robustness

**Figure 6:**
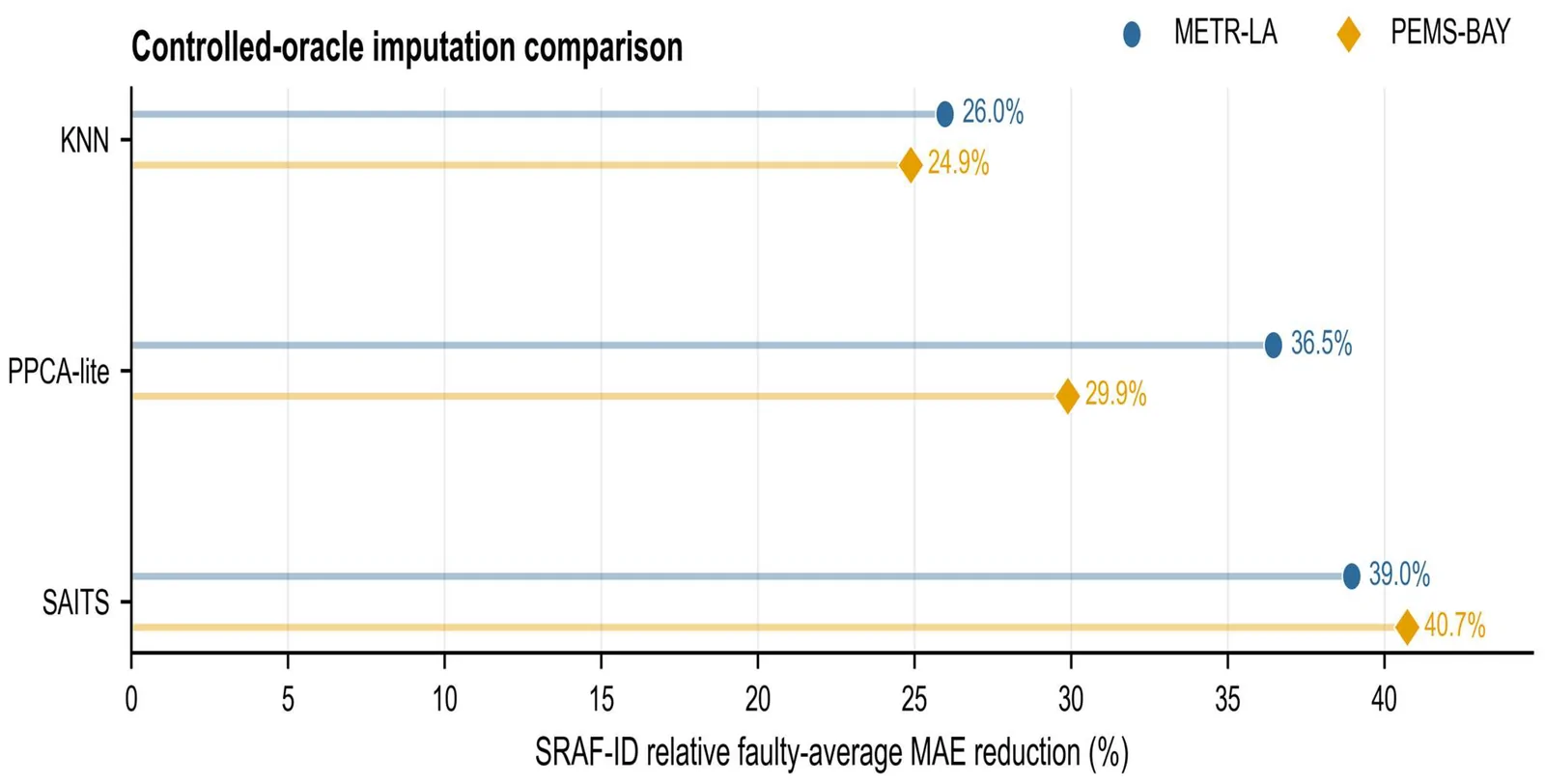
Relative faulty-average MAE reduction of SRAF-ID versus controlled-oracle imputation-then-forecasting pipelines. Values are descriptive downstream comparisons because the imputation pipelines use offline fault locations.

Figure 6 compares SRAF-ID with controlled-oracle KNN+ID-MLP, PPCA-lite+ID-MLP, and PyPOTS-SAITS+ID-MLP pipelines on the same raw-time-disjoint split. SRAF-ID has lower faulty-average forecasting MAE in all six comparisons. Relative reductions range from 24.88% against KNN on PEMS-BAY to 40.72% against PyPOTS-SAITS on PEMS-BAY. Because the imputation pipelines receive the offline fault positions, these results are descriptive downstream comparisons rather than a matched-information ranking of imputation quality.

KNN, PPCA-lite, and PyPOTS-SAITS use the controlled fault-location record to define test-time imputation positions. The resulting imputed test histories are then supplied to their respective ID-MLP forecasters, which were trained on clean training histories. Revised SRAF-ID uses neither finite-valued fault-location labels nor an auxiliary repair loss during final training or inference; it performs repair using observation availability, local variation, and candidate disagreement. Figure 6 therefore compares downstream forecasting error under different information boundaries and should not be interpreted as a general ranking of historical imputation quality.

**Figure 7:**
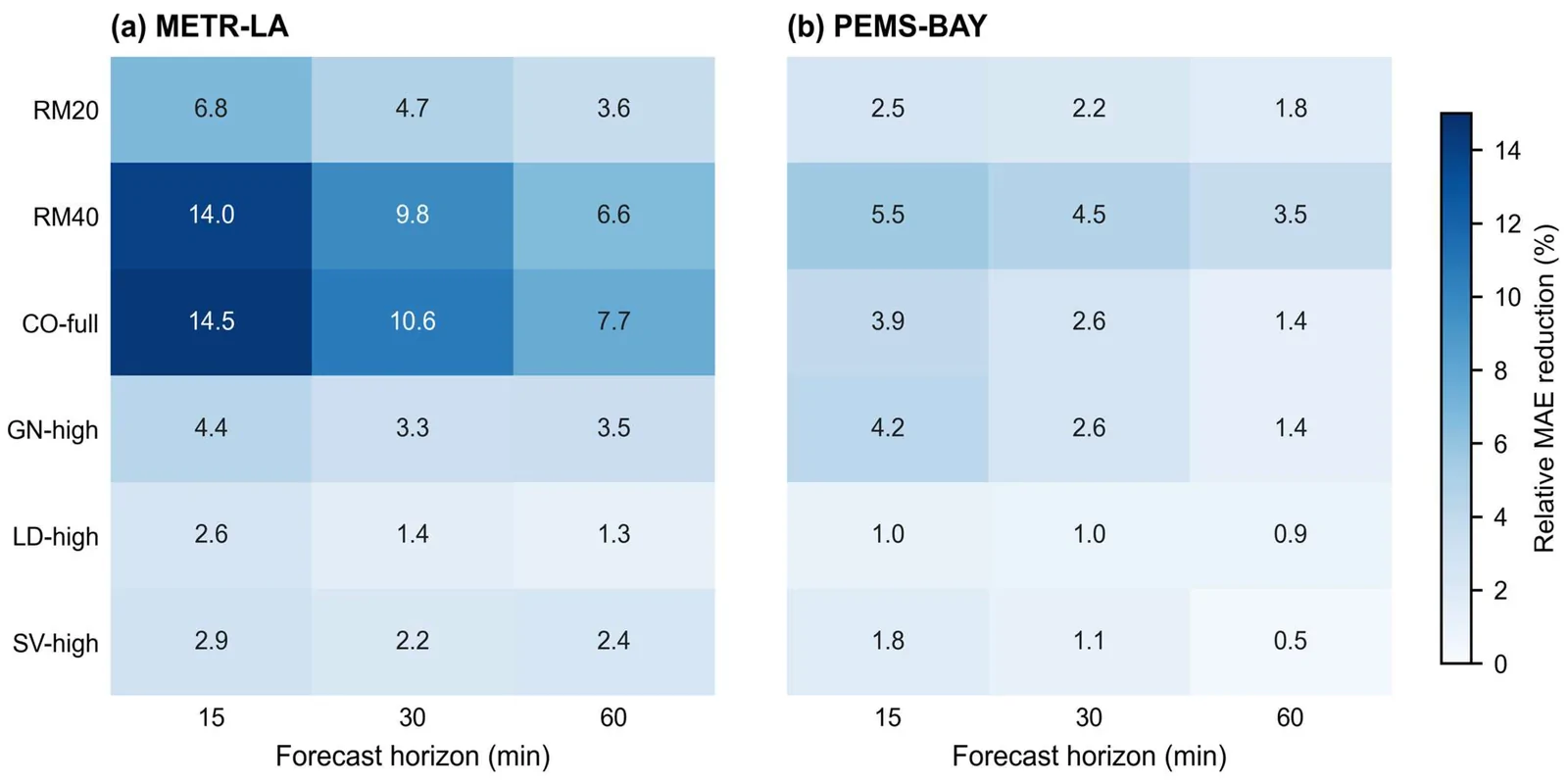
Horizon-specific relative MAE reduction of SRAF-ID over ID-MLP-CA across six controlled perturbations. Columns correspond to 15, 30, and 60 min forecasting horizons.

To examine how robustness changes with forecast lead time, Figure 7 reports relative MAE reductions at horizons 3, 6, and 12, corresponding to 15, 30, and 60 min ahead. Supplementary Table S6 provides the corresponding means, sample standard deviations, paired intervals, and seed-win counts.

At horizon 12, SRAF-ID retains positive mean MAE reductions in all 12 dataset-fault combinations. The smallest final-horizon reductions occur for PEMS-BAY SV-high (0.46%) and LD-high (0.95%), reinforcing the conclusion that improvements under finite-valued perturbations are modest rather than universal or large.

### 4.7. Ablation Study

**Figure 8:**
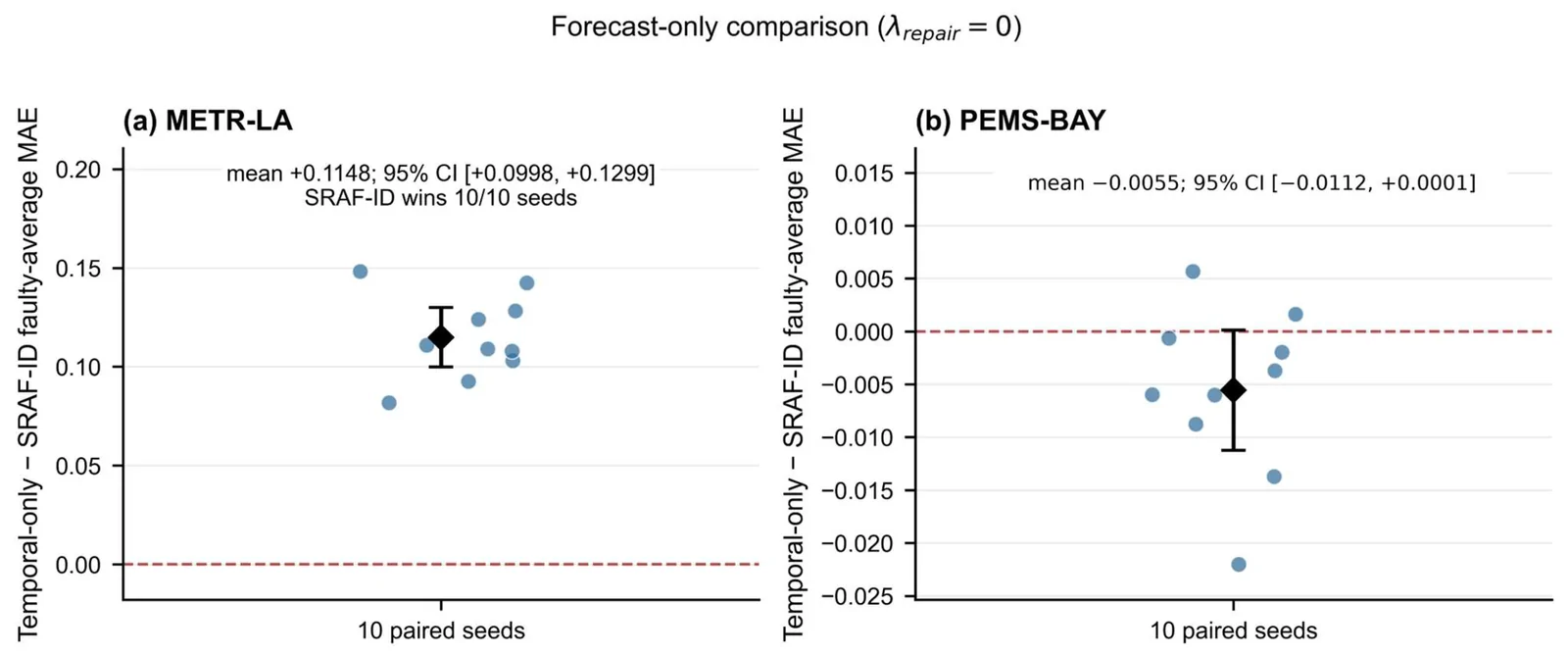
Paired faulty-average MAE difference between Temporal-only and complete SRAF-ID under the same forecast-only objective (λrepair = 0). Positive values favor SRAF-ID; circles show seed-level differences, and diamonds and error bars denote the paired mean and 95% confidence interval.

Figure 8 and Table 4 compare complete SRAF-ID with Temporal-only under the final forecast-only objective (λ_repair_ = 0), the same raw-time-disjoint split, and ten paired seeds. On METR-LA, Temporal-only has 0.1148 higher faulty-average MAE (2.38%), with a paired 95% interval [0.0998, 0.1299], and SRAF-ID is lower in 10/10 seeds. On PEMS-BAY, Temporal-only has 0.0055 lower faulty-average MAE (0.29%), but the interval [−0.0112, 0.0001] includes zero; under clean input, Temporal-only is also 0.0069 lower with interval [−0.0122, −0.0016]. The complete architecture is retained as a unified cross-dataset model because its spatial candidate provides a clear METR-LA benefit while its PEMS-BAY faulty-average difference is small and inconclusive. We do not claim dataset-wise optimality. The broader five-variant matrix under the historical common λ_repair_ = 0.05 objective is reported separately in Supplementary Table S10.

### 4.8. Auxiliary Repair-Loss Analysis

The original submission used λ_repair_ = 0.05 as a heuristic intended to weakly supervise the repair branch while keeping the forecasting loss dominant; it was not derived from a field model or claimed to be optimal. Under the raw-time-disjoint split, we compare λ_repair_ = 0 and 0.05 over ten matched seeds. Mean checkpoint-selection validation scores are 0.22121 versus 0.22127 on METR-LA and 0.20839 versus 0.20861 on PEMS-BAY; these are the composite standardized-space scores defined in Section 4.3. Table 5 shows faulty-average differences (0.05 minus 0) of +0.0045 with 95% CI [−0.0100, 0.0190] on METR-LA and −0.0007 with CI [−0.0040, 0.0026] on PEMS-BAY. Neither dataset shows a stable benefit, so the final model uses λ_repair_ = 0 rather than claiming that 0.05 is justified.

### 4.9. Unseen-Severity and Localized-Fault Evaluation

To test whether the matched gains depend on the exact training perturbations, the frozen λ_repair_ = 0 SRAF-ID and ID-MLP-CA checkpoints were evaluated without retraining under eight additional conditions. These comprise global Gaussian noise and linear drift at 0.25 and 0.75 training-set standard deviations, 5%-sensor-localized noise and drift at 0.50 standard deviations, one-sensor full-window outage, and one-sensor six-step stuck values. Supplementary Table S11 reports all-sensor and affected-sensor statistics, and Figure S6 visualizes paired relative reductions.

For the four localized conditions on each dataset, SRAF-ID has lower mean all-sensor and affected-sensor MAE in all eight dataset-condition pairs. All-sensor reductions range from 0.80% to 2.56%, while affected-sensor reductions range from 1.26% to 43.33%; the largest value occurs for a one-sensor full-history outage on METR-LA. Under unseen global noise, reductions remain positive at both 0.25 and 0.75 standard deviations. Global drift is more difficult: the 0.25-standard-deviation condition remains positive, whereas the 0.75 condition yields mean changes of −1.83% on METR-LA and −1.05% on PEMS-BAY, with paired confidence intervals crossing zero. These checkpoint-only results extend the evidence beyond exact severity and scope matching, but they remain synthetic window-level tests rather than field-event validation.

### 4.10. Computational Overhead

SRAF-ID adds only approximately 0.17% trainable parameters to ID-MLP-CA and is therefore compact in parameter and model-storage terms. In this study, lightweight refers primarily to this compact trainable-parameter and storage footprint rather than latency equivalence with ID-MLP-CA. Parameter counts exclude the carry-forward temporal recurrence, adjacency-based spatial aggregation, ten-feature construction, masking, softmax weighting, blending, intermediate activations, memory traffic, and repeated kernel launches introduced by the repair front-end. These largely parameter-free operations explain why a small parameter increase can coexist with a larger inference-time increase, although no operator-level profiler was used to assign an exact share to each operation. The ratio is also magnified by the unusually small latency of ID-MLP-CA: for batch size 1, the baseline requires only 1.001 ms on METR-LA and 1.175 ms on PEMS-BAY, so absolute increases of approximately 3.39 ms and 3.76 ms correspond to ratios of 4.39× and 4.20×. SRAF-ID itself requires 4.395 ms and 4.932 ms, respectively. For batch size 64, its end-to-end latency is 7.428 ms on METR-LA and 12.930 ms on PEMS-BAY, corresponding to 3.64× and 4.64× the matched baseline. Accordingly, SRAF-ID is parameter-efficient but not latency-neutral.

End-to-end timing includes CPU tensor construction, host-to-device transfer, model forward execution, and output transfer to CPU, while excluding disk loading, fault generation, and checkpoint loading. Each setting uses 50 warm-up iterations, 200 CUDA-synchronized timed iterations, and five independent repetitions. On the tested RTX 4060 Laptop GPU, mean batch-size-one end-to-end latency is 4.395 ms for METR-LA and 4.932 ms for PEMS-BAY; the corresponding P95 values are 4.971 and 5.443 ms. Although the relative ratios are large, the resulting approximately 3–4 ms absolute increments are operationally negligible for the five-minute forecasting cadence used by both datasets. Together with the compact model footprint, this supports practical centralized or network-level inference on the tested hardware and lowers the model-storage barrier for resource-conscious platforms. It does not by itself establish latency, memory, or energy suitability on CPU-only, embedded, or roadside edge hardware; those settings require platform-specific profiling and implementation optimization.

## 5. Discussion

On the raw-time-disjoint split, prediction-oriented repair improves faulty-average MAE by 5.743% on METR-LA and 2.404% on PEMS-BAY, with lower faulty-average MAE in all ten seeds and lower mean MAE in all 12 dataset-fault comparisons. Clean-input MAE also decreases by 2.082% and 0.796%, respectively, showing that robustness is not obtained by sacrificing clean-input forecasting accuracy. The largest reductions occur for METR-LA RM40 and CO-full, both exceeding 10%. Clean-test adjacency diagnostics are consistent with the smaller PEMS-BAY gains: mean off-diagonal edge correlation is 0.7612 on METR-LA and 0.5950 on PEMS-BAY, with 0.86% and 18.24% of edges below 0.3. These post hoc statistics do not prove causality; lower baseline error, traffic heterogeneity, and training dynamics may also contribute.

PEMS-BAY LD-high illustrates why a positive forecasting mean does not imply uniformly effective repair. Because linear drift remains finite and locally smooth, Ω^obs^ does not flag it as unavailable. In standardized space, the graph-neighborhood candidate has higher controlled-position error (0.4655) than the temporal candidate (0.2500), and fusion/final repair errors are 0.2684/0.2585. Nevertheless, downstream MAE is lower by only 0.97%. The repair front-end can therefore improve the prediction objective even when it does not minimize historical reconstruction error; the observed-input blend and spatial branch are not universally optimal for gradual drift.

Compared with controlled-oracle imputation pipelines, SRAF-ID optimizes the explicit repair front-end solely through future forecasting error, directly aligning repair with the downstream task and avoiding a separate historical-reconstruction target. Under the final λ_repair_ = 0 objective, Temporal-only has 2.38% higher faulty-average MAE than complete SRAF-ID on METR-LA, with a paired interval wholly above zero. Temporal-only is 0.29% lower on PEMS-BAY faulty input, but the paired interval includes zero, and it is modestly better under clean input. These results justify retaining one cross-dataset architecture while rejecting any claim that temporal-spatial fusion is universally or dataset-wise optimal. Supplementary Table S10 shows that spatial-only and fixed fusion are clearly weaker under the historical common λ_repair_ = 0.05 objective.

From an application perspective, SRAF-ID can operate as a pre-forecasting module for congestion warning, network monitoring, and emergency traffic organization under degraded sensor histories. It adds only approximately 0.17% trainable parameters. On the tested RTX 4060 Laptop GPU, mean batch-size-one end-to-end latency is 4.395 ms on METR-LA and 4.932 ms on PEMS-BAY, with P95 values of 4.971 and 5.443 ms, respectively. The means are small relative to the five-minute observation interval and support practical network-level inference on this hardware. Relative latency is nevertheless 3.64×–4.64× that of the exceptionally fast ID-MLP-CA baseline at batch size 64 because the repair front-end introduces largely parameter-free tensor, masking, aggregation, and memory operations. SRAF-ID is therefore parameter-efficient and practically fast in the tested setting but not latency-neutral; CPU-only and resource-constrained deployment require separate profiling and optimization.

Several limitations should be emphasized. First, all fault experiments remain synthetic stress tests. Although the fault mechanisms are operationally motivated and additional severity and localization conditions were evaluated, their numerical parameters are heuristic stress-test settings rather than field-calibrated representations of real-world fault frequency, duration, magnitude, or spatial extent. The reported robustness therefore applies to the defined synthetic stress envelope, and validation using field-recorded fault logs remains necessary. Second, the no-repair ID-MLP-CA comparison is not a strict repair-only ablation because SRAF-ID receives the runtime-visible observation mask, whereas ID-MLP-CA does not. For missing and outage conditions, the measured difference may therefore reflect both access to observation availability and the subsequent repair transformation; this confounding is less relevant to finite-valued noise, drift, and stuck-value perturbations, for which the observation mask remains one and does not identify fault locations. Third, the complete temporal-spatial fusion architecture does not consistently outperform Temporal-only on PEMS-BAY. The complete model is retained because of its clear METR-LA benefit and as a unified cross-dataset design, not because fusion is universally or dataset-wise optimal. Current evidence is also limited to two public sensor networks and one identity-enhanced forecasting backbone.

## 6. Conclusions

This paper proposed SRAF-ID, a prediction-oriented speed-channel repair framework for faulty historical observations. It constructs a same-sensor temporal candidate and a mask-aware graph-neighborhood candidate from the supplied off-diagonal adjacency, combines them through learned two-way softmax fusion, and preserves node identity and periodic temporal context through a bypass pathway. The complete repair-and-forecasting pipeline is trained end to end using future forecasting loss only, so the final model does not require fault-location labels during training or inference.

Across two raw-time-disjoint public datasets, six window-level perturbations, and ten matched seeds, SRAF-ID reduces faulty-average MAE by 5.7% on METR-LA and 2.4% on PEMS-BAY relative to a corruption-aware same-backbone baseline. It achieves lower mean MAE in all 12 dataset-fault comparisons and lower faulty-average MAE in all ten seeds on both datasets, while clean-input MAE improves by 2.1% and 0.8%. In checkpoint-only distribution-shift tests, all eight localized dataset-condition pairs retain positive mean gains for both all-sensor and affected-sensor MAE. Unseen 0.75-standard-deviation global drift is the clearest boundary: mean gains are −1.83% and −1.05%, with paired intervals crossing zero. The matched METR-LA LD-high RMSE increase is also retained.

Taken together, the evidence establishes SRAF-ID as a compact, prediction-oriented, and fault-label-free repair front-end that improves future forecasting under the matched held-out protocol and remains beneficial for the tested localized shifts without retraining. The final-objective ablation shows a clear complete-model advantage on METR-LA and a small, inconclusive faulty-input difference on PEMS-BAY, defining the contribution as a unified cross-dataset design rather than a universally optimal fusion rule. The approximately 0.17% parameter increase and mean batch-size-one latencies of 4.395 ms and 4.932 ms support practical use on the tested GPU; the corresponding P95 values are 4.971 and 5.443 ms. These conclusions remain bounded by the synthetic, heuristically parameterized fault protocol and the observation-mask confounding in the no-repair comparison. Future work should therefore prioritize field-calibrated fault evaluation, a mask-preserving no-repair baseline, broader cross-backbone validation, adaptive handling of severe drift, and lower-latency implementation.

## Figures and Tables

**Table 1:** Dataset characteristics and forecasting protocol.

Dataset	Number of Sensors	Historical Window L	Prediction Horizon H	Fault Application	Prediction Target	Formal Seeds
METR-LA	207	12	12	historical input speed only	clean future speed	42–51
PEMS-BAY	325	12	12	historical input speed only	clean future speed	42–51

**Table 2:** Controlled faulty-observation settings and model-visible information.

Fault Setting	Abbrev.	Key Parameter	Model-Visible Input	Offline Fault Record
random missing 20%	RM20	*p* = 0.20	missing entries; Ω^obs^ = 0	audit; diagnostics; seed statistics; controlled-oracle imputation
random missing 40%	RM40	*p* = 0.40	missing entries; Ω^obs^ = 0	audit; diagnostics; seed statistics; controlled-oracle imputation
continuous outage	CO-full	sensor rate = 0.10; L = 12 (24-step request truncated)	outage entries; Ω^obs^ = 0	audit; diagnostics; seed statistics; controlled-oracle imputation
Gaussian noise	GN-high	σ = 0.50 *σ*_train_	finite values; Ω^obs^ = 1	audit and diagnostics only; not a model input
linear drift	LD-high	ramp 0 → 0.50 *σ*_train_	finite values; Ω^obs^ = 1	audit and diagnostics only; not a model input
stuck value	SV-high	sensor rate = 0.50; length = 6	finite values; Ω^obs^ = 1	audit and diagnostics only; not a model input

Note: RM = random missing, CO = continuous outage, GN = Gaussian noise, LD = linear drift, and SV = stuck value. Ω^obs^ denotes the model-visible observation mask. M^fault^ denotes an offline controlled-protocol record used for auditing, diagnostics, seed-level statistics, and controlled-oracle imputation; it is not used to train or run SRAF-ID.

**Table 3:** Faulty- and clean-input MAE of matched ID-MLP-CA and SRAF-ID.

Dataset	Input Setting	ID-MLP-CA MAE	SRAF-ID MAE	Relative MAE Reduction
METR-LA	Faulty average	5.1173 ± 0.0394	4.8234 ± 0.0137	5.743%
METR-LA	Clean	4.7341 ± 0.0274	4.6355 ± 0.0145	2.082%
PEMS-BAY	Faulty average	1.9918 ± 0.0096	1.9439 ± 0.0059	2.404%
PEMS-BAY	Clean	1.8171 ± 0.0097	1.8026 ± 0.0037	0.796%

Note: Values are mean ± sample standard deviation across ten seeds. For faulty input, each seed is first averaged across the six test faults before cross-seed statistics are computed. Positive relative reduction favors SRAF-ID.

**Table 4:** Forecast-only comparison of complete SRAF-ID and Temporal-only.

Dataset	Scope	SRAF-ID MAE	Temporal-Only MAE	Temporal-Only-SRAF-ID (95% CI)
METR-LA	Faulty average	4.8234 ± 0.0137	4.9382 ± 0.0193	+0.1148 [0.0998, 0.1299]
METR-LA	Clean	4.6355 ± 0.0145	4.6486 ± 0.0178	+0.0131 [0.0007, 0.0254]
PEMS-BAY	Faulty average	1.9439 ± 0.0059	1.9383 ± 0.0058	−0.0055 [−0.0112, 0.0001]
PEMS-BAY	Clean	1.8026 ± 0.0037	1.7957 ± 0.0069	−0.0069 [−0.0122, −0.0016]

Note: Values are mean ± sample standard deviation across ten matched seeds. Differences are Temporal-only MAE minus SRAF-ID MAE; positive values favor SRAF-ID. Faulty-average values are computed within-seed across six perturbations before cross-seed statistics. Both models use λ_repair_ = 0.

**Table 5:** Auxiliary repair-loss ablation under an otherwise identical SRAF-ID architecture.

Dataset	λ = 0 Faulty MAE	λ = 0.05 Faulty MAE	Difference (0.05–0)	Paired 95% CI
METR-LA	4.8234 ± 0.0137	4.8279 ± 0.0142	+0.0045	[−0.0100, 0.0190]
PEMS-BAY	1.9439 ± 0.0059	1.9432 ± 0.0057	−0.0007	[−0.0040, 0.0026]

Note: Values are mean ± sample standard deviation across ten seed-level six-fault averages. Positive differences indicate higher MAE with the auxiliary loss. Validation-only sensitivity results are reported in Supplementary Table S8.

## Data Availability

The METR-LA and PEMS-BAY datasets analyzed in this study are publicly available through Zenodo at https://doi.org/10.5281/zenodo.5146275 (accessed on 13 August 2026). An accompanying SRAF-ID implementation and compact evidence archive is available as GitHub Release v1.1.0 at https://github.com/PengLuis/sensor-reliability-aware-traffic-monitoring/releases/tag/v1.1.0 (accessed on 13 August 2026); Release v1.0.2 is retained for original-submission provenance. The revised raw-time-disjoint audit artifacts, full per-seed logs, and checkpoints are retained by the authors and are available from the corresponding author on reasonable request. Raw and processed datasets, checkpoints, and large logs are not redistributed in the public archive.

## Supplementary Materials

The following supporting information can be downloaded at: https://www.mdpi.com/article/10.3390/s26165263/s1, Figure S1: Mean learned temporal and graph-neighborhood fusion weights across the six controlled perturbations. Each stacked bar sums to one; Figure S2: Repair-candidate errors across dataset-fault settings; Figure S3: Raw-time-disjoint split audit and window counts; Figure S4: PEMS-BAY LD-high candidate quality under the raw-time-disjoint protocol; Figure S5: Dataset-level candidate-quality comparison; Figure S6: Checkpoint-only severity- and localization-shift evaluation. Markers denote all-sensor and affected-sensor relative MAE reductions; error bars are paired 95% confidence intervals over ten seeds, and positive values favor SRAF-ID; Table S1: Seed-level paired MAE statistics for the matched comparison; Table S2: RMSE robustness results; Table S3: Fault-wise MAE robustness results; Table S4: Parameter count and end-to-end batch inference latency; Table S5: Imputation-then-forecasting comparison; Table S6: Horizon-specific MAE results at 15, 30, and 60 minutes; Table S7: Auxiliary repair-loss ablation; Table S8: Repair-loss-weight sensitivity; Table S9: Candidate-quality and adjacency-correlation diagnostics; Table S10: Historical common-objective architecture matrix; Table S11: Checkpoint-only evaluation under unseen severities and localized window-level faults.
