# Supplementary material for: SRAF-ID: A Sensor-Reliability-Aware Framework for Robust Traffic Speed Forecasting Under Missing and Faulty Sensor Observations
Source: Sensors (Basel). 2026 Aug 19;26(16):5263. doi: 10.3390/s26165263 (PMC13517813; doi:10.3390/s26165263)
Supplement: Supplementary file 1 [file sensors-26-05263-s001.zip › sensors-4412261-supplementary-revised.pdf]

sensors

MDPI

Supplementary Materials

SRAF-ID: A Sensor-Reliability-Aware Framework for Robust Traffic Speed Forecasting under Missing and Faulty Sensor Observations

Peng Lu 1,2,3, Daming Wu 2,3,*, Shaofei Lan 2,3, Beinan Guo 2 and Zixiao Li 2

1 School of Emergency Management Science and Engineering, University of Chinese Academy of Sciences, Beijing 100049, China; lupeng24@mails.ucas.ac.cn (P.L.)
2 National Institute of Natural Hazards, Ministry of Emergency Management of the People's Republic of China, Beijing 100085, China; lanshaofei24@mails.ucas.ac.cn (S.L.); 202131260004@mail.bnu.edu.cn (B.G.); zixiaoli@ninhm.ac.cn (Z.L.)
3 Key Laboratory of Compound and Chained Natural Hazards Dynamics, Ministry of Emergency Management of China, Beijing 100085, China
* Correspondence: damingwu@ninhm.ac.cn (D.W.)

Supplementary Tables

All main, fault-wise, horizon, imputation, auxiliary-loss, architecture, distribution-shift, and diagnostic results use the raw-time-disjoint split and ten formal seeds (42-51). The final SRAF-ID, no-repair baseline, and primary Temporal-only comparison use λ_repair = 0. Tables S7-S8 isolate the auxiliary objective; Table S10 retains the broader historical architecture matrix under common λ_repair = 0.05; and Table S11 reports checkpoint-only unseen-severity and localized-fault tests. Sample standard deviations use ddof = 1; paired 95% t intervals are descriptive and are not adjusted for family-wise multiplicity.

Table S1. Seed-level paired MAE statistics for the matched comparison.

Dataset	Fault	n	Mean delta (SRAF-ID - ID-MLP-CA)	95% CI	SRAF-ID seed wins	Paired t p	Wilcoxon p	Interpretation
METR-LA	RM20	10	-0.2448	[-0.2706, -0.2189]	10	4.99e-09	0.00506	SRAF-ID lower mean MAE
METR-LA	RM40	10	-0.5436	[-0.5898, -0.4974]	10	7.23e-10	0.00506	SRAF-ID lower mean MAE
METR-LA	CO-full	10	-0.5884	[-0.6085, -0.5684]	10	2e-13	0.00506	SRAF-ID lower mean MAE

 https://doi.org/10.3390/xxxxx

Dataset	Fault	n	Mean delta (SRAF-ID - ID-MLP-CA)	95% CI	SRAF-ID seed wins	Paired t p	Wilcoxon p	Interpretation
METR-LA	GN-high	10	-0.1818	[-0.2117, -0.1518]	10	2.41e-07	0.00506	SRAF-ID lower mean MAE
METR-LA	LD-high	10	-0.0841	[-0.1302, -0.0379]	9	0.00259	0.00934	SRAF-ID lower mean MAE
METR-LA	SV-high	10	-0.1208	[-0.1440, -0.0976]	10	8.96e-07	0.00506	SRAF-ID lower mean MAE
PEMS-BAY	RM20	10	-0.0415	[-0.0488, -0.0342]	10	4.33e-07	0.00506	SRAF-ID lower mean MAE
PEMS-BAY	RM40	10	-0.0937	[-0.1040, -0.0834]	10	7.25e-09	0.00506	SRAF-ID lower mean MAE
PEMS-BAY	CO-full	10	-0.0528	[-0.0631, -0.0425]	10	1.05e-06	0.00506	SRAF-ID lower mean MAE
PEMS-BAY	GN-high	10	-0.0592	[-0.0668, -0.0516]	10	2.64e-08	0.00506	SRAF-ID lower mean MAE
PEMS-BAY	LD-high	10	-0.0197	[-0.0316, -0.0078]	9	0.00461	0.0125	SRAF-ID lower mean MAE
PEMS-BAY	SV-high	10	-0.0205	[-0.0288, -0.0121]	10	0.000369	0.00506	SRAF-ID lower mean MAE

Mean delta is SRAF-ID MAE minus ID-MLP-CA MAE; negative values favor SRAF-ID. All 12 MAE means are negative under the corrected raw-time-disjoint protocol, while Table S2 retains the adverse METR-LA LD-high RMSE result.

Table S2. RMSE robustness results.

Dataset	Fault	ID-MLP-CA RMSE mean ± SD	SRAF-ID RMSE mean ± SD	Relative RMSE reduction	Direction	Seeds
METR-LA	RM20	12.4321 ± 0.0631	12.2177 ± 0.0230	1.72%	SRAF-ID lower	10
METR-LA	RM40	12.9603 ± 0.0841	12.4170 ± 0.0230	4.19%	SRAF-ID lower	10
METR-LA	CO-full	13.6551 ± 0.0615	12.3905 ± 0.0217	9.26%	SRAF-ID lower	10
METR-LA	GN-high	12.4472 ± 0.0664	12.2164 ± 0.0187	1.85%	SRAF-ID lower	10
METR-LA	LD-high	12.2607 ± 0.0561	12.3542 ± 0.0403	-0.76%	SRAF-ID higher	10
METR-LA	SV-high	12.5311 ± 0.0819	12.4202 ± 0.0284	0.88%	SRAF-ID lower	10
PEMS-BAY	RM20	4.2484 ± 0.0216	4.1763 ± 0.0102	1.70%	SRAF-ID lower	10
PEMS-BAY	RM40	4.4720 ± 0.0296	4.3103 ± 0.0126	3.61%	SRAF-ID lower	10
PEMS-BAY	CO-full	4.5196 ± 0.0261	4.2984 ± 0.0071	4.89%	SRAF-ID lower	10
PEMS-BAY	GN-high	4.4370 ± 0.0152	4.3519 ± 0.0058	1.92%	SRAF-ID lower	10
PEMS-BAY	LD-high	4.3974 ± 0.0195	4.3652 ± 0.0232	0.73%	SRAF-ID lower	10
PEMS-BAY	SV-high	4.2935 ± 0.0338	4.2505 ± 0.0134	1.00%	SRAF-ID lower	10

Positive relative reduction indicates lower RMSE for SRAF-ID. Values are mean ± sample standard deviation across ten matched seeds.

Table S3. Fault-wise MAE robustness results.

Dataset	Fault	ID-MLP-CA MAE mean ± SD	SRAF-ID MAE mean ± SD	Relative MAE reduction	95% CI of SRAF-ID - baseline	SRAF-ID seed wins	Direction
METR-LA	RM20	4.9563 ± 0.0380	4.7115 ± 0.0150	4.94%	[-0.2706, -0.2189]	10	SRAF-ID lower
METR-LA	RM40	5.3851 ± 0.0685	4.8415 ± 0.0154	10.09%	[-0.5898, -0.4974]	10	SRAF-ID lower
METR-LA	CO-full	5.4201 ± 0.0282	4.8317 ± 0.0148	10.86%	[-0.6085, -0.5684]	10	SRAF-ID lower
METR-LA	GN-high	5.0088 ± 0.0442	4.8270 ± 0.0124	3.63%	[-0.2117, -0.1518]	10	SRAF-ID lower
METR-LA	LD-high	5.0135 ± 0.0524	4.9294 ± 0.0238	1.68%	[-0.1302, -0.0379]	9	SRAF-ID lower
METR-LA	SV-high	4.9199 ± 0.0384	4.7991 ± 0.0152	2.46%	[-0.1440, -0.0976]	10	SRAF-ID lower
PEMS-BAY	RM20	1.8914 ± 0.0105	1.8500 ± 0.0046	2.19%	[-0.0488, -0.0342]	10	SRAF-ID lower
PEMS-BAY	RM40	2.0177 ± 0.0151	1.9240 ± 0.0053	4.64%	[-0.1040, -0.0834]	10	SRAF-ID lower
PEMS-BAY	CO-full	1.9960 ± 0.0148	1.9432 ± 0.0042	2.65%	[-0.0631, -0.0425]	10	SRAF-ID lower
PEMS-BAY	GN-high	2.1357 ± 0.0077	2.0765 ± 0.0070	2.77%	[-0.0668, -0.0516]	10	SRAF-ID lower
PEMS-BAY	LD-high	2.0245 ± 0.0133	2.0048 ± 0.0138	0.97%	[-0.0316, -0.0078]	9	SRAF-ID lower
PEMS-BAY	SV-high	1.8853 ± 0.0127	1.8648 ± 0.0042	1.09%	[-0.0288, -0.0121]	10	SRAF-ID lower

Positive relative reduction indicates lower MAE for SRAF-ID. Confidence intervals are for SRAF-ID minus ID-MLP-CA; intervals below zero favor SRAF-ID.

Table S4. Parameter count and end-to-end batch inference latency.

Dataset	Batch size	ID-MLP-CA params	SRAF-ID params	Parameter increase	ID-MLP-CA E2E latency mean ± SD (ms)	SRAF-ID E2E latency mean ± SD (ms)	SRAF-ID P95 (ms)	Latency ratio	SRAF-ID throughput (samples/s)
METR-LA	1	117,868	118,078	0.178%	1.001 ± 0.094	4.395 ± 0.292	4.971	4.39×	227.5
METR-LA	64	117,868	118,078	0.178%	2.043 ± 0.143	7.428 ± 0.445	8.412	3.64×	8,615.6
PEMS-BAY	1	121,644	121,854	0.173%	1.175 ± 0.205	4.932 ± 0.390	5.443	4.20×	202.8
PEMS-BAY	64	121,644	121,854	0.173%	2.784 ± 0.194	12.930 ± 0.509	13.844	4.64×	4,949.6

End-to-end timing includes CPU tensor construction, host-to-device transfer, model forward execution, and output transfer to CPU; it excludes disk loading, fault generation, and checkpoint loading. The benchmark used 50 warm-up iterations, 200 synchronized timed iterations, and five repetitions. Forward-only timing is not used as a paper-facing metric.

Table S5. Imputation-then-forecasting comparison.

Dataset	Comparison model	Comparison MAE mean ± SD	SRAF-ID MAE mean ± SD	SRAF-ID relative reduction	95% CI of SRAF-ID - comparison	SRAF-ID seed wins	Information boundary
METR-LA	KNN+ID-MLP	6.5159 ± 0.0766	4.8234 ± 0.0137	25.98%	[-1.7477, -1.6373]	10	controlled-oracle fault positions
METR-LA	PPCA-lite+ID-MLP	7.5904 ± 0.0376	4.8234 ± 0.0137	36.45%	[-2.7986, -2.7355]	10	controlled-oracle fault positions
METR-LA	PyPOTS-SAITS+ID-MLP	7.9009 ± 0.0954	4.8234 ± 0.0137	38.95%	[-3.1481, -3.0070]	10	controlled-oracle fault positions
PEMS-BAY	KNN+ID-MLP	2.5876 ± 0.0152	1.9439 ± 0.0059	24.88%	[-0.6550, -0.6324]	10	controlled-oracle fault positions
PEMS-BAY	PPCA-lite+ID-MLP	2.7726 ± 0.0140	1.9439 ± 0.0059	29.89%	[-0.8399, -0.8176]	10	controlled-oracle fault positions
PEMS-BAY	PyPOTS-SAITS+ID-MLP	3.2792 ± 0.1575	1.9439 ± 0.0059	40.72%	[-1.4508, -1.2199]	10	controlled-oracle fault positions

The comparison pipelines use controlled-oracle fault positions to define imputation locations. SRAF-ID does not use finite-valued fault-location labels during training or inference; therefore, the information boundaries differ.

Table S6. Horizon-specific MAE results at 15, 30, and 60 minutes.

Dataset	Input setting	Horizon	ID-MLP-CA MAE mean ± SD	SRAF-ID MAE mean ± SD	Relative MAE reduction	95% CI of SRAF-ID - baseline	SRAF-ID seed wins
METR-LA	Clean	H3	3.7340 ± 0.0289	3.6430 ± 0.0194	2.44%	[-0.1084, -0.0736]	10
METR-LA	Clean	H6	4.7634 ± 0.0225	4.6780 ± 0.0139	1.79%	[-0.1017, -0.0692]	10
METR-LA	Clean	H12	6.1315 ± 0.0430	6.0041 ± 0.0135	2.08%	[-0.1585, -0.0963]	10
METR-LA	RM20	H3	4.0177 ± 0.0300	3.7451 ± 0.0196	6.78%	[-0.2918, -0.2534]	10
METR-LA	RM20	H6	4.9841 ± 0.0314	4.7511 ± 0.0145	4.67%	[-0.2549, -0.2110]	10
METR-LA	RM20	H12	6.2693 ± 0.0565	6.0463 ± 0.0135	3.56%	[-0.2616, -0.1845]	10
METR-LA	RM40	H3	4.5473 ± 0.0634	3.9124 ± 0.0184	13.96%	[-0.6786, -0.5912]	10
METR-LA	RM40	H6	5.4035 ± 0.0606	4.8750 ± 0.0161	9.78%	[-0.5709, -0.4861]	10
METR-LA	RM40	H12	6.5632 ± 0.0873	6.1310 ± 0.0139	6.59%	[-0.4909, -0.3735]	10
METR-LA	CO-full	H3	4.5542 ± 0.0263	3.8950 ± 0.0181	14.47%	[-0.6760, -0.6424]	10
METR-LA	CO-full	H6	5.4431 ± 0.0231	4.8648 ± 0.0158	10.62%	[-0.5954, -0.5612]	10
METR-LA	CO-full	H12	6.6378 ± 0.0434	6.1293 ± 0.0130	7.66%	[-0.5398, -0.4771]	10
METR-LA	GN-high	H3	4.0712 ± 0.0441	3.8936 ± 0.0142	4.36%	[-0.2066, -0.1486]	10
METR-LA	GN-high	H6	5.0180 ± 0.0408	4.8545 ± 0.0110	3.26%	[-0.1920, -0.1348]	10
METR-LA	GN-high	H12	6.3348 ± 0.0561	6.1161 ± 0.0143	3.45%	[-0.2572, -0.1802]	10
METR-LA	LD-high	H3	4.1213 ± 0.0351	4.0162 ± 0.0274	2.55%	[-0.1413, -0.0689]	9
METR-LA	LD-high	H6	5.0444 ± 0.0497	4.9719 ± 0.0259	1.44%	[-0.1194, -0.0258]	9
METR-LA	LD-high	H12	6.2497 ± 0.0787	6.1712 ± 0.0252	1.26%	[-0.1381, -0.0188]	9
METR-LA	SV-high	H3	3.9734 ± 0.0362	3.8594 ± 0.0166	2.87%	[-0.1354, -0.0926]	10
METR-LA	SV-high	H6	4.9429 ± 0.0371	4.8353 ± 0.0148	2.18%	[-0.1294, -0.0860]	10
METR-LA	SV-high	H12	6.2595 ± 0.0542	6.1109 ± 0.0163	2.38%	[-0.1839, -0.1134]	10
PEMS-BAY	Clean	H3	1.4803 ± 0.0070	1.4621 ± 0.0031	1.23%	[-0.0227, -0.0137]	10
PEMS-BAY	Clean	H6	1.8494 ± 0.0089	1.8342 ± 0.0045	0.82%	[-0.0220, -0.0084]	10
PEMS-BAY	Clean	H12	2.3143 ± 0.0155	2.3046 ± 0.0059	0.42%	[-0.0212, 0.0018]	7
PEMS-BAY	RM20	H3	1.5659 ± 0.0125	1.5265 ± 0.0040	2.52%	[-0.0470, -0.0320]	10
PEMS-BAY	RM20	H6	1.9176 ± 0.0106	1.8758 ± 0.0052	2.18%	[-0.0492, -0.0344]	10
PEMS-BAY	RM20	H12	2.3739 ± 0.0102	2.3319 ± 0.0063	1.77%	[-0.0505, -0.0334]	10
PEMS-BAY	RM40	H3	1.7186 ± 0.0190	1.6237 ± 0.0049	5.52%	[-0.1066, -0.0831]	10
PEMS-BAY	RM40	H6	2.0337 ± 0.0154	1.9429 ± 0.0058	4.47%	[-0.1012, -0.0804]	10
PEMS-BAY	RM40	H12	2.4651 ± 0.0106	2.3781 ± 0.0070	3.53%	[-0.0962, -0.0778]	10

Dataset	Input setting	Horizon	ID-MLP-CA MAE mean ± SD	SRAF-ID MAE mean ± SD	Relative MAE reduction	95% CI of SRAF-ID - baseline	SRAF-ID seed wins
PEMS-BAY	CO-full	H3	1.7195 ± 0.0156	1.6521 ± 0.0040	3.92%	[-0.0774, -0.0572]	10
PEMS-BAY	CO-full	H6	2.0217 ± 0.0148	1.9682 ± 0.0052	2.65%	[-0.0637, -0.0433]	10
PEMS-BAY	CO-full	H12	2.4159 ± 0.0158	2.3825 ± 0.0053	1.38%	[-0.0452, -0.0217]	10
PEMS-BAY	GN-high	H3	1.8896 ± 0.0072	1.8106 ± 0.0069	4.18%	[-0.0852, -0.0729]	10
PEMS-BAY	GN-high	H6	2.1440 ± 0.0078	2.0878 ± 0.0073	2.62%	[-0.0636, -0.0488]	10
PEMS-BAY	GN-high	H12	2.5088 ± 0.0113	2.4737 ± 0.0074	1.40%	[-0.0461, -0.0241]	10
PEMS-BAY	LD-high	H3	1.7521 ± 0.0141	1.7339 ± 0.0152	1.04%	[-0.0313, -0.0050]	8
PEMS-BAY	LD-high	H6	2.0426 ± 0.0161	2.0224 ± 0.0124	0.99%	[-0.0313, -0.0090]	8
PEMS-BAY	LD-high	H12	2.4218 ± 0.0092	2.3988 ± 0.0161	0.95%	[-0.0360, -0.0100]	9
PEMS-BAY	SV-high	H3	1.5704 ± 0.0104	1.5423 ± 0.0049	1.79%	[-0.0345, -0.0218]	10
PEMS-BAY	SV-high	H6	1.9122 ± 0.0119	1.8916 ± 0.0049	1.08%	[-0.0288, -0.0125]	10
PEMS-BAY	SV-high	H12	2.3564 ± 0.0180	2.3457 ± 0.0059	0.46%	[-0.0228, 0.0013]	7

H3, H6, and H12 correspond to 15, 30, and 60 minutes ahead. Confidence intervals are paired across ten seeds.

Table S7. Auxiliary repair-loss ablation.

Dataset	Scope	λ = 0 mean ± SD	λ = 0.05 mean ± SD	Difference (0.05 - 0)	95% CI	λ = 0.05 seed wins	Interpretation
METR-LA	Faulty average	4.8234 ± 0.0137	4.8279 ± 0.0142	+0.0045	[-0.0100, 0.0190]	3	No stable auxiliary-loss benefit
METR-LA	Clean	4.6355 ± 0.0145	4.6357 ± 0.0136	+0.0002	[-0.0137, 0.0141]	3	No stable auxiliary-loss benefit
PEMS-BAY	Faulty average	1.9439 ± 0.0059	1.9432 ± 0.0057	-0.0007	[-0.0040, 0.0026]	4	No stable auxiliary-loss benefit
PEMS-BAY	Clean	1.8026 ± 0.0037	1.8024 ± 0.0044	-0.0002	[-0.0027, 0.0023]	4	No stable auxiliary-loss benefit

Positive differences indicate higher MAE when the auxiliary repair loss is used. The complete repair architecture is identical; only the training objective differs. The historical auxiliary term measures $|X\_rep - X|$ only at controlled fault positions ($M\_fault = 1$), divides their sum by $\max(1, \Sigma\, M\_fault)$, and is added to the forecasting loss after multiplication by λ_repair.

Table S8. Repair-loss-weight sensitivity.

Dataset	λ	n	Checkpoint-selection validation score (standardized space)	Checkpoint-selection score 95% CI	Clean test MAE (inverse-transformed speed scale)	Faulty-average test MAE (inverse-transformed speed scale)	Selection interpretation
METR-LA	0.00	10	0.2212 ± 0.0004	[0.2209, 0.2215]	4.6355 ± 0.0145	4.8234 ± 0.0137	Final objective
METR-LA	0.05	10	0.2213 ± 0.0003	[0.2211, 0.2215]	4.6357 ± 0.0136	4.8279 ± 0.0142	Original auxiliary objective
PEMS-BAY	0.00	10	0.2084 ± 0.0005	[0.2080, 0.2088]	1.8026 ± 0.0037	1.9439 ± 0.0059	Final objective
PEMS-BAY	0.05	10	0.2086 ± 0.0005	[0.2082, 0.2090]	1.8024 ± 0.0044	1.9432 ± 0.0057	Original auxiliary objective

The checkpoint-selection validation score is reported in standardized model space. It equals 0.5 times clean validation MAE plus 0.5 times the mean of MAE on fixed RM40, GN-high, and SV-high validation corruptions, and it is used for early stopping and checkpoint selection. Clean and faulty test MAEs are inverse-transformed to the original speed scale (miles per hour, mph) and should not be compared numerically with the validation score. The table reports the two objectives available under the corrected raw-time-disjoint protocol. Differences between λ_repair = 0 and 0.05 are small, and the paired test evidence in Table S7 does not show a stable auxiliary-loss benefit.

Table S9. Candidate-quality and adjacency-correlation diagnostics.

Panel A. Adjacency-edge speed-correlation statistics.

Dataset	Mean correlation	Median correlation	Interquartile range	Edges with correlation < 0.3
METR-LA	0.7612	0.7817	[0.6606, 0.8995]	0.86%
PEMS-BAY	0.5950	0.6661	[0.4102, 0.8254]	18.24%

Panel B. PEMS-BAY LD-high candidate diagnostics at controlled fault positions (standardized model space).

Representation	MAE mean ± SD (standardized model space)	Interpretation
Temporal candidate	0.2500 ± 0.0000	Same-sensor carry-forward candidate
Graph-neighborhood candidate	0.4655 ± 0.0000	Higher error under gradual drift
Fused candidate	0.2684 ± 0.0011	Learned two-way fusion
Final repaired history	0.2585 ± 0.0005	After observed-input blend

These post hoc diagnostics were not used for model or hyperparameter selection and do not establish causality. Adjacency correlations use the reconstructed clean raw-time-disjoint test stream; unit self-loops are excluded and Pearson correlation is summarized over every directed nonzero off-diagonal supplied-adjacency edge. Candidate-quality metrics are calculated at controlled fault positions over the complete held-out test set and all ten forecast-only SRAF-ID seeds in standardized model space.

Table S10. Historical common-objective architecture matrix.

Dataset	Variant	Faulty-input MAE	Difference vs. full	Interpretation
METR-LA	SRAF-ID	4.8279 ± 0.0142	+0.000%	Two-way softmax fusion
METR-LA	Sigmoid-gate fusion	4.8329 ± 0.0167	+0.104%	Single sigmoid gate
METR-LA	Temporal-only	4.9382 ± 0.0193	+2.285%	Temporal candidate only
METR-LA	Spatial-only	5.2609 ± 0.0596	+8.970%	Graph-neighborhood candidate only
METR-LA	Fixed fusion	4.9564 ± 0.0506	+2.663%	Fixed 0.5/0.5 fusion
PEMS-BAY	SRAF-ID	1.9432 ± 0.0057	+0.000%	Two-way softmax fusion
PEMS-BAY	Sigmoid-gate fusion	1.9446 ± 0.0067	+0.073%	Single sigmoid gate
PEMS-BAY	Temporal-only	1.9383 ± 0.0058	-0.250%	Temporal candidate only
PEMS-BAY	Spatial-only	2.1348 ± 0.0039	+9.861%	Graph-neighborhood candidate only
PEMS-BAY	Fixed fusion	2.0029 ± 0.0067	+3.073%	Fixed 0.5/0.5 fusion

This secondary matrix holds λ_repair = 0.05 common across five variants and is retained for component context. The primary final-objective comparison in Table 4 retrains Temporal-only with λ_repair = 0. Sigmoid-gate fusion uses one learned sigmoid coefficient α for αXtemp + (1-α)Xsp, whereas SRAF-ID uses a learned two-way softmax.

Table S11. Checkpoint-only evaluation under unseen severities and localized window-level faults.

Dataset	Condition	Metric scope	ID-MLP-CA MAE	SRAF-ID MAE	Relative reduction	Paired 95% CI	SRAF-ID wins
METR-LA	GN-global-0.25σ	All sensors	4.8042	4.6828	+2.53%	[0.1008, 0.1420]	10/10
METR-LA	GN-global-0.25σ	Affected sensors	4.8042	4.6828	+2.53%	[0.1008, 0.1420]	10/10
METR-LA	GN-global-0.75σ	All sensors	5.4585	5.1124	+6.34%	[0.2910, 0.4012]	10/10
METR-LA	GN-global-0.75σ	Affected sensors	5.4585	5.1124	+6.34%	[0.2910, 0.4012]	10/10
METR-LA	LD-global-0.25σ	All sensors	4.8054	4.7345	+1.48%	[0.0459, 0.0959]	10/10
METR-LA	LD-global-0.25σ	Affected sensors	4.8054	4.7345	+1.48%	[0.0459, 0.0959]	10/10
METR-LA	LD-global-0.75σ	All sensors	5.4408	5.5406	-1.83%	[-0.2074, 0.0080]	2/10
METR-LA	LD-global-0.75σ	Affected sensors	5.4408	5.5406	-1.83%	[-0.2074, 0.0080]	2/10
METR-LA	GN-local-5%-0.50σ	All sensors	4.7474	4.6447	+2.16%	[0.0837, 0.1217]	10/10
METR-LA	GN-local-5%-0.50σ	Affected sensors	5.0038	4.8155	+3.76%	[0.1637, 0.2128]	10/10
METR-LA	LD-local-5%-0.50σ	All sensors	4.7475	4.6468	+2.12%	[0.0809, 0.1205]	10/10

Dataset	Condition	Metric scope	ID-MLP-CA MAE	SRAF-ID MAE	Relative reduction	Paired 95% CI	SRAF-ID wins
METR-LA	LD-local-5%-0.50σ	Affected sensors	5.0090	4.8663	+2.85%	[0.0958, 0.1895]	10/10
METR-LA	CO-single-sensor-full	All sensors	4.7670	4.6448	+2.56%	[0.1032, 0.1411]	10/10
METR-LA	CO-single-sensor-full	Affected sensors	11.5311	6.5348	+43.33%	[4.9115, 5.0812]	10/10
METR-LA	SV-single-sensor-6	All sensors	4.7359	4.6371	+2.09%	[0.0799, 0.1176]	10/10
METR-LA	SV-single-sensor-6	Affected sensors	5.0926	4.9300	+3.19%	[0.1250, 0.2002]	10/10
PEMS-BAY	GN-global-0.25σ	All sensors	1.9834	1.9260	+2.90%	[0.0491, 0.0658]	10/10
PEMS-BAY	GN-global-0.25σ	Affected sensors	1.9834	1.9260	+2.90%	[0.0491, 0.0658]	10/10
PEMS-BAY	GN-global-0.75σ	All sensors	2.3508	2.2659	+3.61%	[0.0733, 0.0966]	10/10
PEMS-BAY	GN-global-0.75σ	Affected sensors	2.3508	2.2659	+3.61%	[0.0733, 0.0966]	10/10
PEMS-BAY	LD-global-0.25σ	All sensors	2.0600	2.0501	+0.48%	[0.0040, 0.0159]	9/10
PEMS-BAY	LD-global-0.25σ	Affected sensors	2.0600	2.0501	+0.48%	[0.0040, 0.0159]	9/10
PEMS-BAY	LD-global-0.75σ	All sensors	2.5292	2.5558	-1.05%	[-0.0647, 0.0115]	1/10
PEMS-BAY	LD-global-0.75σ	Affected sensors	2.5292	2.5558	-1.05%	[-0.0647, 0.0115]	1/10
PEMS-BAY	GN-local-5%-0.50σ	All sensors	1.8328	1.8161	+0.91%	[0.0097, 0.0237]	10/10
PEMS-BAY	GN-local-5%-0.50σ	Affected sensors	2.1361	2.0764	+2.79%	[0.0528, 0.0666]	10/10
PEMS-BAY	LD-local-5%-0.50σ	All sensors	1.8273	1.8124	+0.82%	[0.0080, 0.0218]	10/10
PEMS-BAY	LD-local-5%-0.50σ	Affected sensors	2.0230	1.9975	+1.26%	[0.0139, 0.0371]	9/10
PEMS-BAY	CO-single-sensor-full	All sensors	1.8227	1.8070	+0.86%	[0.0086, 0.0228]	10/10
PEMS-BAY	CO-single-sensor-full	Affected sensors	3.6521	3.2406	+11.27%	[0.3610, 0.4621]	10/10
PEMS-BAY	SV-single-sensor-6	All sensors	1.8175	1.8030	+0.80%	[0.0074, 0.0216]	10/10
PEMS-BAY	SV-single-sensor-6	Affected sensors	1.9549	1.9260	+1.48%	[0.0201, 0.0377]	10/10

The frozen λ_repair = 0 SRAF-ID and ID-MLP-CA checkpoints are evaluated without parameter updates. Localized sensor sets are resampled per held-out window. Affected-sensor MAE evaluates future predictions for sensors whose historical window was perturbed. Positive relative reduction and positive baseline-minus-SRAF confidence intervals favor SRAF-ID. Global conditions affect every sensor, so all-sensor and affected-sensor rows coincide.

Supplementary Figures

Figures S1-S5 are regenerated from the raw-time-disjoint matched evidence and provide descriptive diagnostics and a split audit. Figure S6 reports the separately specified checkpoint-only distribution-shift tests. No supplementary figure is used to select the final objective or architecture, and the observed associations are not causal evidence.

Figure S1 summarizes the average learned temporal and graph-neighborhood fusion weights. Larger temporal weights indicate greater reliance on same-sensor continuity, whereas larger graph-neighborhood weights indicate stronger use of visible adjacent-sensor evidence.

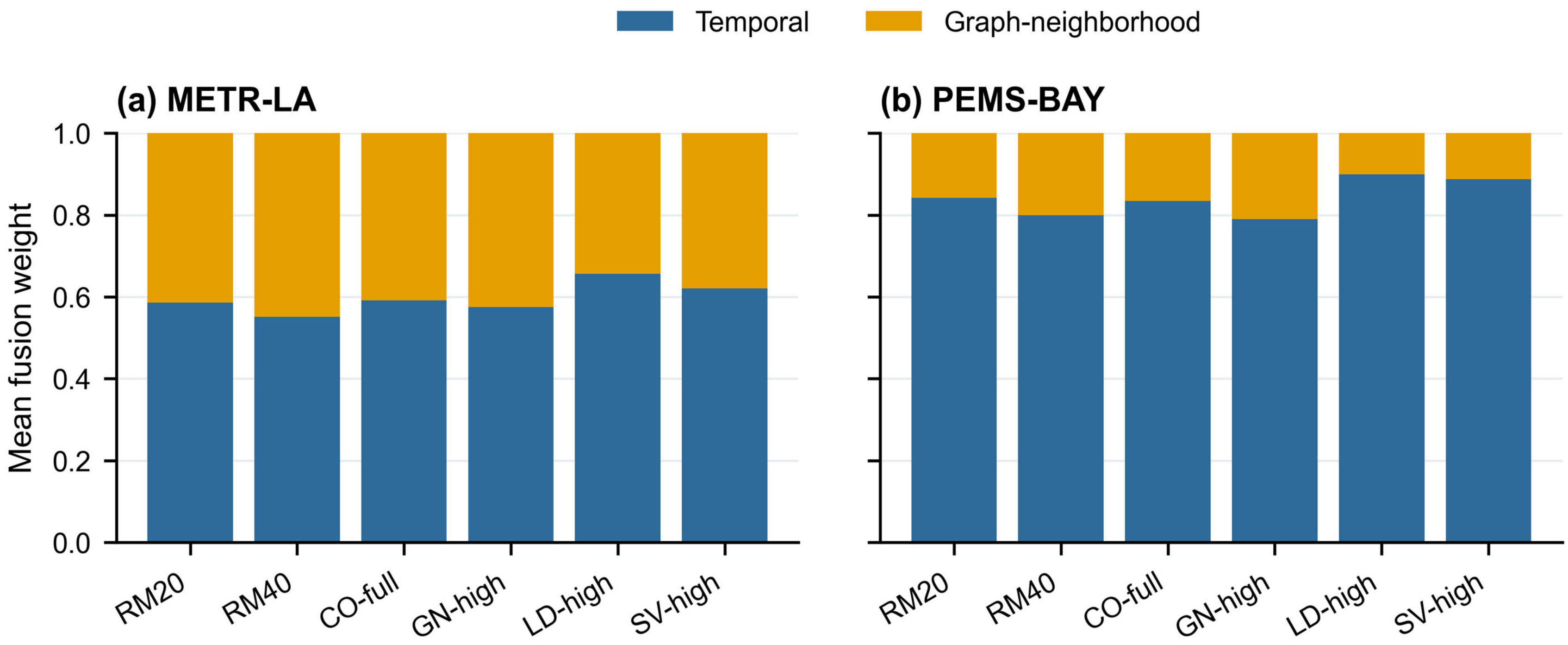

Figure S1. Mean learned temporal and graph-neighborhood fusion weights across the six controlled perturbations. Each stacked bar sums to one.

Figure S2 compares temporal, spatial, fused, and final repaired-history MAE. The comparison illustrates that candidate quality varies by dataset and fault type.

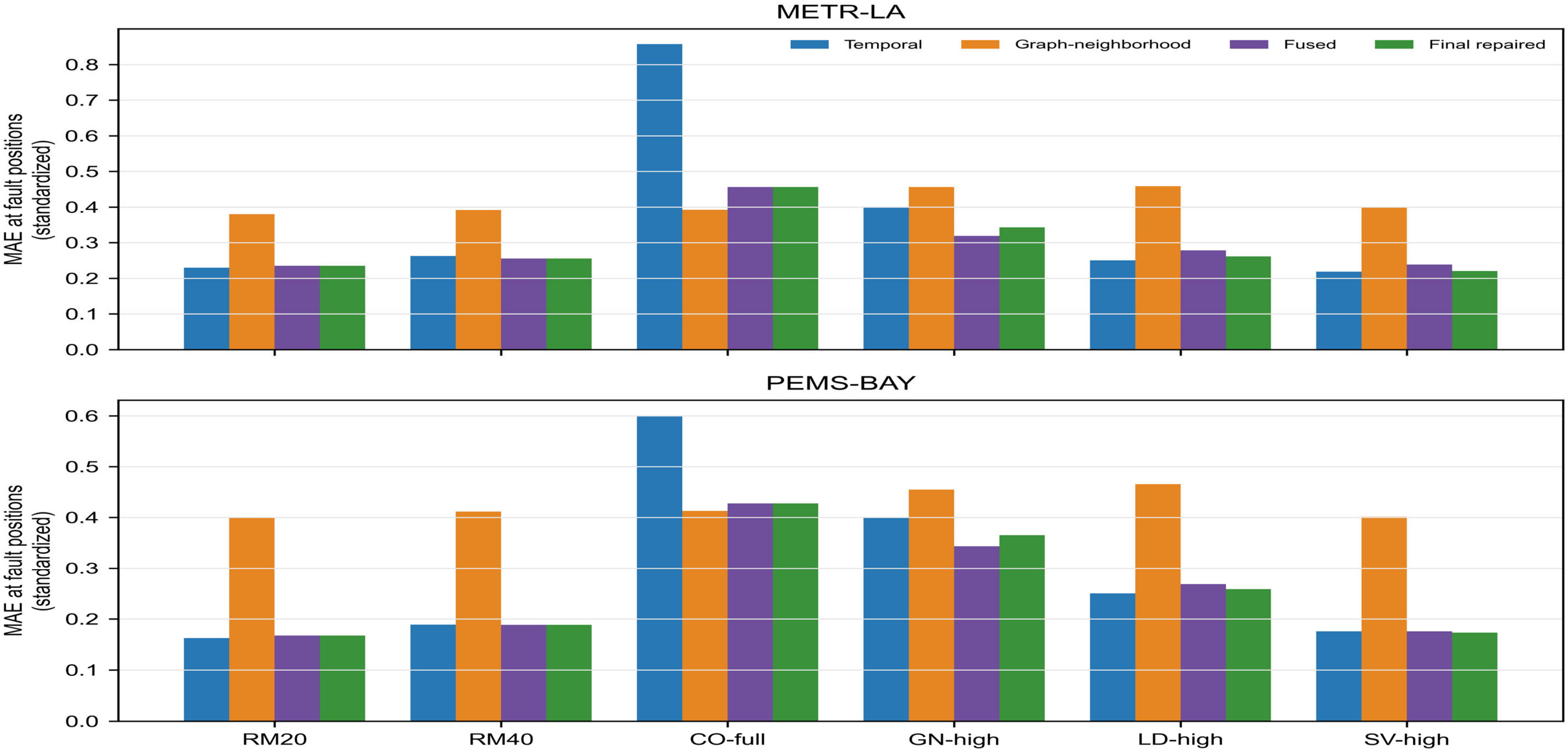

Figure S2. Repair-candidate errors across dataset-fault settings.

Figure S3 reports the train, validation, and test window counts after splitting the raw chronological series before window formation. All audited cross-split raw-time intersections are zero.

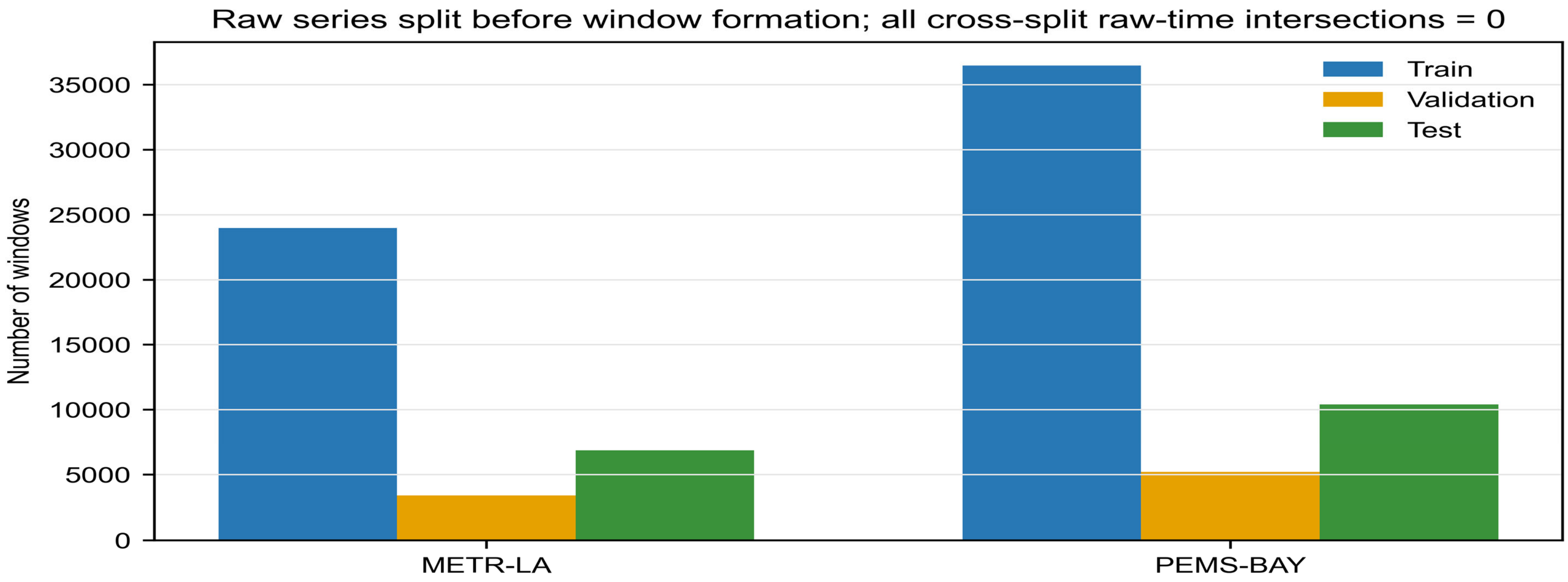

Figure S3. Raw-time-disjoint split audit and window counts.

Figure S4 aggregates PEMS-BAY LD-high candidate errors at all controlled positions over the complete held-out test set and ten forecast-only seeds. The graph-neighborhood candidate is less accurate than the temporal candidate in standardized space, even though downstream forecast MAE is modestly lower than the matched baseline.

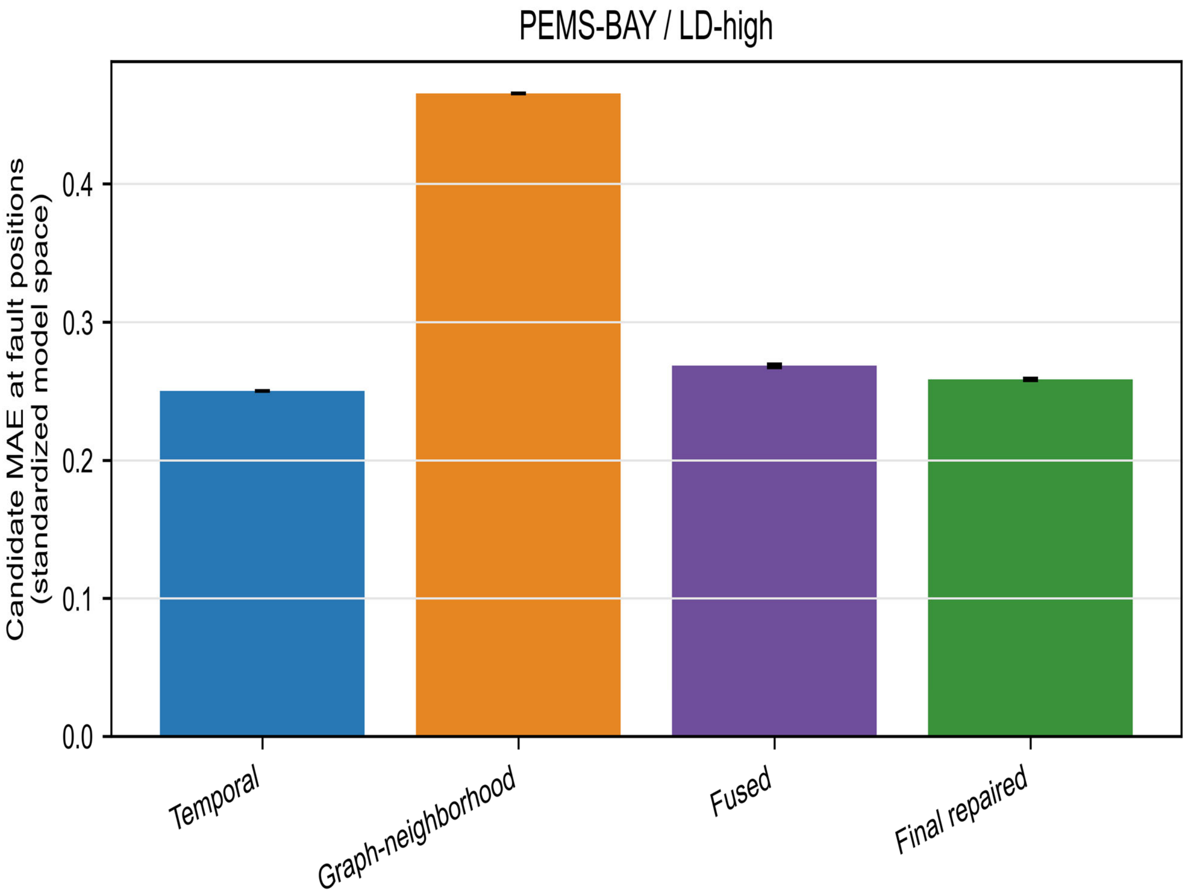

Figure S4. PEMS-BAY LD-high candidate quality under the raw-time-disjoint protocol.

Figure S5 averages candidate error over the six controlled perturbations within each dataset. Values are in standardized model space and are descriptive rather than a cross-dataset speed-unit comparison.

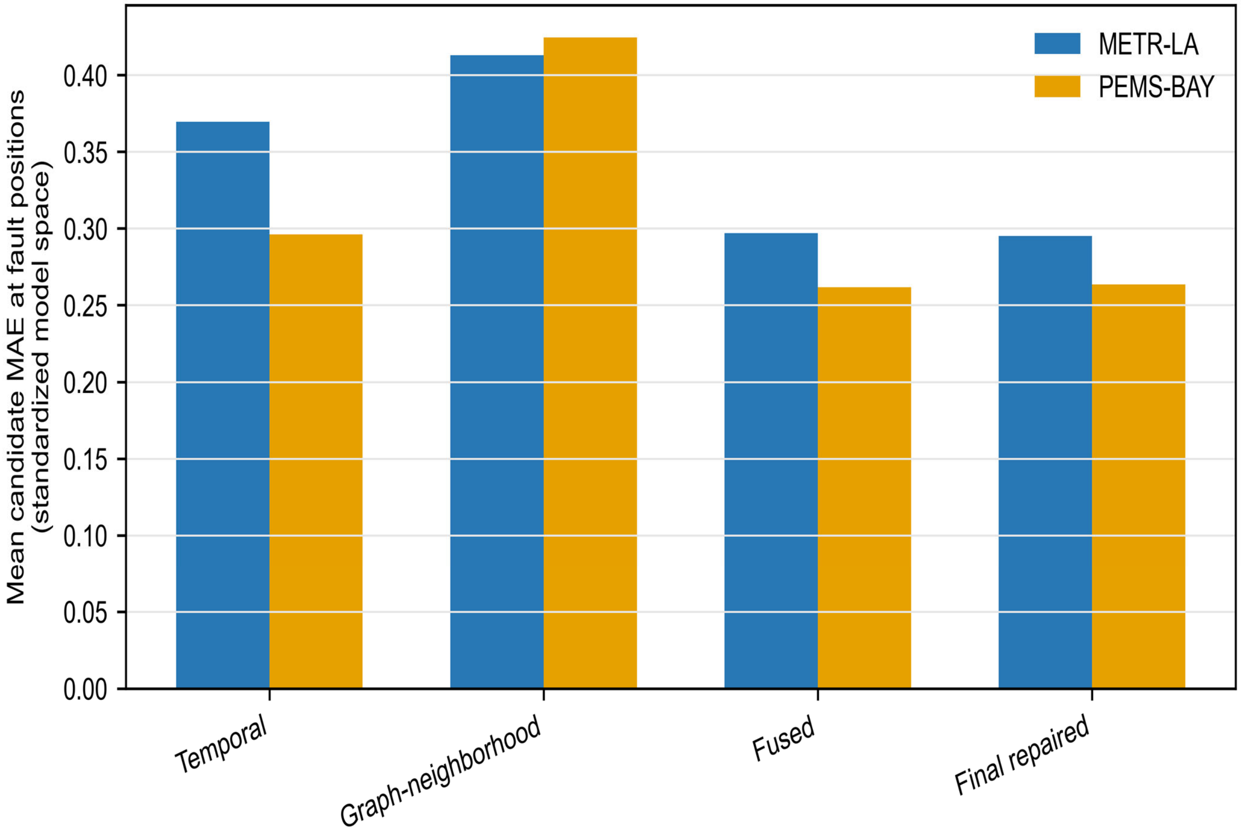

Figure S5. Dataset-level candidate-quality comparison.

Figure S6 compares all-sensor and affected-sensor relative MAE reductions under unseen severity and localized per-window conditions. Error bars are paired 95% confidence intervals over ten seeds. Positive values favor SRAF-ID; the global conditions perturb every sensor, so their two markers overlap apart from a small display offset.

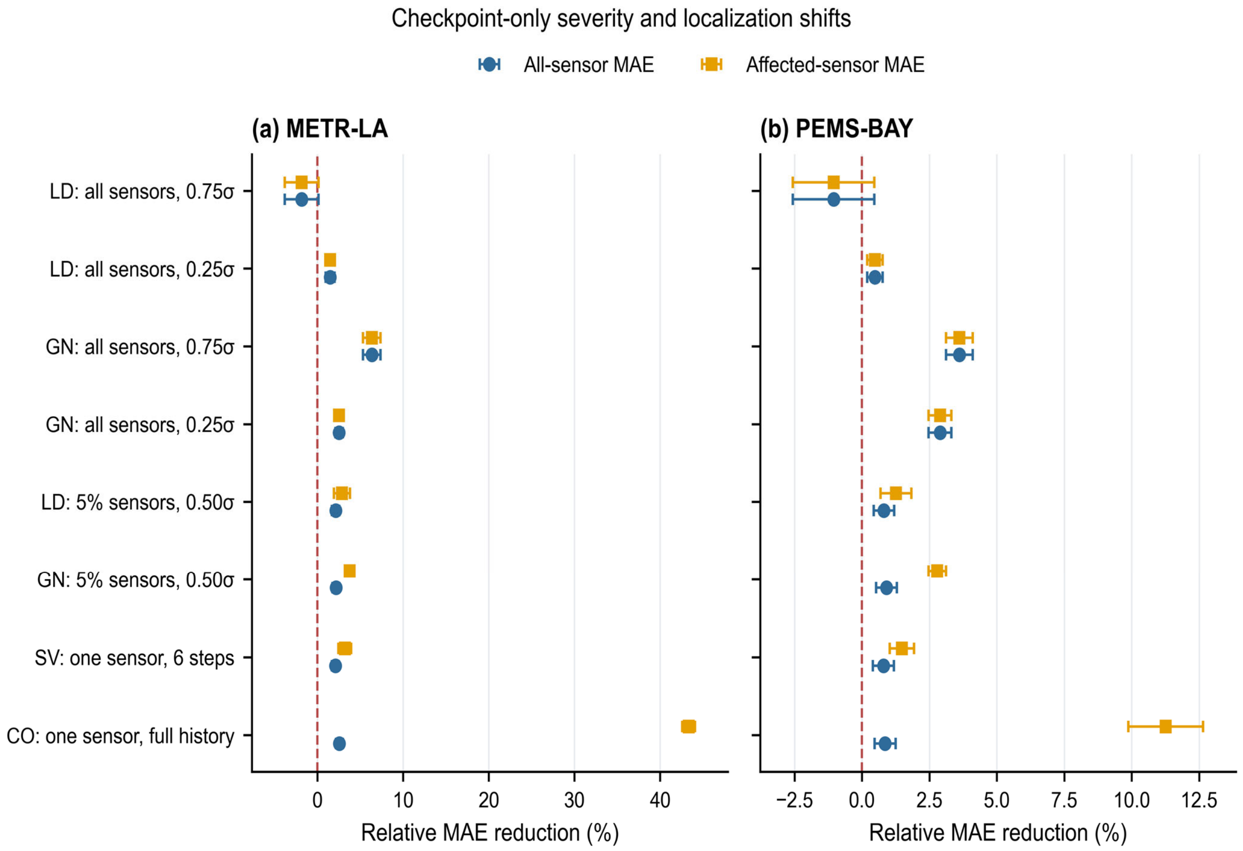

Figure S6. Checkpoint-only severity- and localization-shift evaluation. Markers denote all-sensor and affected-sensor relative MAE reductions; error bars are paired 95% confidence intervals over ten seeds, and positive values favor SRAF-ID.
